# SGL-Mamba: Structure-Aware Global–Local Mamba for Crack Segmentation

**DOI:** 10.3390/s26165040

**Published:** 2026-08-08

**Authors:** Mozi Gao, Zhonghua Weng, Jiasheng Wu, Xiaoman Qi, Guanghui Liao, Qinying Zou, Mengyu Wang

**Affiliations:** 1 School of Land Science and Technology, China University of Geosciences (Beijing), Beijing 100083, China; 2112250078@email.cugb.edu.cn (M.G.); qxm@email.cugb.edu.cn (X.Q.); 3012240021@email.cugb.edu.cn (Q.Z.); 18738321882@163.com (M.W.); 2 Pearl River Water Resources Research Institute, Pearl River Water Resources Commission of the Ministry of Water Resources, Guangzhou 510611, China; wujiasheng99@outlook.com (J.W.); 15917363740@163.com (G.L.)

**Keywords:** crack segmentation, visual state space model, structure-aware modeling, deep learning, attention mechanism

## Abstract

**Highlights:**

**What are the main findings?**

- This paper proposes the SGL-Mamba network, introducing an SGL-Mamba block that combines a parallel directional scanning mechanism with a local perception branch to effectively achieve joint modeling of global topological continuity and local crack details.
- A high–low frequency separation enhancement module is incorporated to adaptively suppress background noise, while a deformable large kernel attention decoder is integrated to dynamically capture and adapt to irregular crack orientations.

**What are the implications of the main findings?**

- Extensive evaluations on Crack500, DeepCrack, and CrackMap datasets demonstrate that SGL-Mamba consistently outperforms existing state-of-the-art methods, exhibiting accurate target delineation quality, structural coherence, and strong robustness against complex backgrounds.
- By successfully balancing a lightweight network architecture with high computational efficiency, this method provides a highly promising and practical solution for automated, high-precision crack detection in routine infrastructure health monitoring and safety assessments.

**Abstract:**

Accurate crack segmentation based on optical sensor imagery is of paramount importance for infrastructure health monitoring and disaster early warning. However, in complex natural scenes, cracks typically exhibit characteristics such as being thin and elongated, multi-branched, and having low contrast. Existing segmentation models struggle to balance low computational overhead with the simultaneous modeling of global topological continuity and the precise extraction of local details. To overcome the aforementioned limitations, we introduce a Structure-Aware Global–Local Mamba (SGL-Mamba). Firstly, the SGL-Mamba Block is designed in the encoding stage, which significantly enhances the joint modeling capacity for continuous topological structures and edge textures through the synergy of a parallel directional scanning mechanism and a local perception branch. Secondly, we design a High–Low Frequency Separation Enhancement (HLFSE) module to reconstruct the skip connections. This module leverages frequency decoupling to adaptively suppress high-frequency background noise and alleviate the semantic gap. Finally, in the decoding stage, the Deformable Large Kernel Attention (D-LKA) is integrated, utilizing a dynamic spatial receptive field to precisely adapt to irregular crack orientations. Extensive experiments on three public datasets (Crack500, DeepCrack, and CrackMap) demonstrate that SGL-Mamba outperforms other state-of-the-art (SOTA) methods, achieving an F1 score (F1) of 0.7982 ± 0.0038 and an mIoU of 0.8011 ± 0.0036 on the Crack500 dataset. While ensuring lightweight architecture and high computational efficiency, the proposed method provides an effective and practical solution for automatic crack detection.

## 1. Introduction

Cracks, as critical indicators of geological disasters, road pavement distress, and structural damage, are of great significance for infrastructure health monitoring, disaster early warning, and structural safety assessment [1,2,3,4]. In complex natural environments, cracks generally present features such as thinness, low contrast, multi-branching, and irregular extensions. Additionally, they show significant sensitivity to environmental artifacts such as shadows, noise, shifting illumination, and intricate background patterns. Therefore, achieving high-precision, pixel-level automatic crack segmentation in complex scenarios is a core objective in spatial imagery analysis and automated monitoring [5].

Early crack detection predominantly relied on traditional image processing techniques, such as thresholding segmentation [6] and wavelet transform methods [7], which extracted crack regions via grayscale, texture, and frequency-domain information. Building upon this, subsequent research incorporated classical pattern recognition models, specifically Support Vector Machines (SVM) [8] and Random Forests (RF) [9], to classify manually designed features, thereby improving detection performance in complex scenes. However, constrained by their reliance on handcrafted feature construction, these methods inherently lack sufficient feature expression capacity and scene adaptability. They often struggle to maintain stable performance when faced with complex backgrounds, illumination changes, and significant variations in crack scale.

With the rapid advancement of deep learning, semantic segmentation models based on convolutional neural networks (CNNs), represented by FCN [10] and PSPNet [11], have achieved remarkable progress in visual scene parsing and have gradually been introduced into the crack detection domain. Compared to traditional handcrafted features, CNNs can automatically extract multi-level semantic features through end-to-end learning, significantly enhancing crack representation capabilities in complex scenes. Driven by this, researchers have proposed a multitude of improved models specifically tailored for thin and elongated crack structures [12,13,14,15]. Nevertheless, the inherent local receptive fields and spatial inductive biases of convolutional networks limit their effectiveness in modeling global dependencies across long-range pixels. Consequently, they remain prone to discontinuous segmentation and inadequate background noise suppression in scenes with complex topological structures and large-scale, irregular cracks. To further bolster global context modeling, visual Transformer architectures, such as Vision Transformer (ViT) [16], Swin Transformer [17], and SegFormer [18], have been successively proposed and widely applied to crack extraction tasks [2,3,19]. By establishing semantic associations between distant pixels via self-attention mechanisms, Transformers model the global topological continuity of fine crack structures more effectively. However, the computational complexity of self-attention grows quadratically with input resolution. This leads to substantial memory consumption and computational overhead in high-resolution tasks.

Recently, the Mamba architecture has attracted extensive attention by offering linear scaling in computation while maintaining an outstanding capability to capture long-distance contextual dependencies [20,21,22]. As research deepens, an increasing number of works have begun to extend Mamba to various downstream visual tasks [23,24,25,26]. For crack segmentation, Mamba’s global sequence modeling capability inherently strengthens the long-range topological associations among thin crack structures, thereby improving the overall connectivity modeling of complex crack regions. However, crack targets typically feature thin extensions, curved bifurcations, and complex directional distributions, posing higher demands on the 2D sequence modeling capabilities of existing visual Mamba models. Currently, most Mamba-based methods employ fixed-direction or parallel cross-scanning strategies to serialize 2D features. This rearrangement can disrupt spatial neighborhood relationships, weakening the model’s perception of crack directional continuity and topology [27,28,29]. Additionally, the original Mamba architecture lacks the local spatial inductive bias inherent to CNNs; thus, its ability to delineate fine-grained edge textures and local structural details remains limited in scenarios involving complex backgrounds, blurred boundaries, and low contrast. Therefore, how to further enhance the joint modeling of continuous crack structures, local boundary details, and robustness against complex backgrounds without compromising global modeling efficiency remains a critical challenge in current crack segmentation tasks.

To address the aforementioned issues, this paper proposes the SGL-Mamba network. First, we design the SGL-Mamba Block in the encoding stage. Distinct from conventional sequential state-space modules, it uniquely introduces a dual-pathway architecture, which enhances the sequential modeling of continuous crack topologies via a parallel directional scanning mechanism, while concurrently employing a dedicated local perception branch to fortify the model’s feature awareness of fine-grained edge textures and local details, compensating for the lack of spatial inductive bias in pure Mamba models. Second, to distinguish our decoding strategy from previous U-shaped Mamba architectures, we design the HLFSE module in the skip connections, utilizing frequency separation and dynamic modulation strategies to amplify high-frequency edge information while suppressing background noise propagation, effectively mitigating the semantic gap in U-shape networks. Finally, we construct a joint decoding framework incorporating D-LKA in the decoding stage. By leveraging dynamic spatial receptive field modeling, this framework enhances adaptability to irregular crack structures and blurred boundary regions, achieving high-precision segmentation in complex crack scenarios.

In summary, the main contributions of this paper are as follows:

(1) Recognizing Mamba as an established sequence modeling technique, we propose the SGL-Mamba Block, which, through the synergistic modeling of a global structure-aware pathway and a local perception pathway, enhances the network’s ability to model continuous crack topologies and directional dependencies while maintaining high computational efficiency.
(2) Building upon the existing concepts of frequency enhancement and deformable attention, we design the HLFSE module to strengthen crack edge responses via frequency separation and dynamic modulation, effectively alleviating the semantic gap and background noise propagation in the skip connections of U-shape networks. Concurrently, a D-LKA decoder is utilized to enhance spatial modeling for complex boundaries and irregular crack regions.
(3) To verify the model’s effectiveness, thorough experiments are carried out using three public datasets: Crack500, DeepCrack, and CrackMap. Empirical evaluations indicate that the introduced framework outperforms existing mainstream approaches regarding target delineation quality, structural coherence, and adaptability within highly cluttered scenes to complex backgrounds, validating its effectiveness in scenarios with complex topological structures and low contrast.

## 2. Related Work

### 2.1. Crack Segmentation Methods

Prior to the advent of deep learning, crack extraction frameworks were fundamentally based on traditional handcrafted feature extraction and image processing techniques. Although simple to implement, these methods are highly dependent on manually designed features and prior knowledge, possessing inherent limitations in feature representation. They are extremely prone to false positives and missed detections in scenes with complex background interference.

With the development of deep learning, CNN-based crack segmentation methods have gradually become the mainstream research direction. Early works were predominantly based on Fully Convolutional Networks (FCNs) and U-Net architectures, achieving end-to-end pixel-level crack region extraction through encoder–decoder structures [10,30]. For instance, Zou et al. [31] proposed DeepCrack, which leverages a hierarchical multi-scale feature fusion strategy to significantly improve the continuity of thin crack structure representation. Qu et al. [13] effectively improved feature learning in complex boundary regions by introducing deep supervision mechanisms and multi-scale feature cascade structures. Bai et al. [14] proposed DEHF-Net, which combined a dual-path encoding structure with an attention fusion mechanism, substantially improving defect segmentation performance under complex backgrounds. Additionally, FPHBN, proposed by Yang et al. [15] significantly enhanced multi-scale detection capabilities for complex pavement cracks by constructing a feature pyramid and a hierarchical boosting module. Fei et al. [32] developed CrackNet-V, which leverages multi-layer feature aggregation and pixel-level prediction to enhance crack feature representation and improve segmentation robustness under complex backgrounds. Deng et al. [33] innovatively integrated the Region Proposal Network (RPN) with deep feature learning to achieve precise localization and detection of crack candidate regions. Although CNN-based methods have significantly improved segmentation accuracy, their inherent local convolution operations and restricted receptive fields hinder the effective modeling of global dependencies across long-range pixels. When processing cracks with complex topological structures and large-scale irregular extensions, issues such as segmentation discontinuities, loss of detail, and inadequate background noise suppression remain prevalent. To overcome these limitations, GFSegNet [2] significantly enhanced global feature modeling for mining area cracks by integrating Fourier convolutions with a hierarchical Transformer structure; HFSV-Net [3] effectively elevated pavement distress recognition in complex scenes using multi-scale feature fusion and multi-task learning strategies. Compared to traditional CNN methods, Transformers can establish semantic associations between pixels at any distance via self-attention mechanisms, thereby modeling the global topological continuity of thin cracks more effectively. However, the computational complexity of their self-attention mechanisms grows quadratically with input resolution, leading to exorbitant computational and memory overhead in high-resolution dense prediction tasks.

Overall, while existing CNN and Transformer methods have achieved favorable performance in crack segmentation, they are still susceptible to boundary fragmentation, missed details, and mis-segmentation in complex backgrounds when dealing with thin, multi-branched, and long-range continuous crack structures. Thus, maintaining efficient modeling while further enhancing the joint representation of crack directional continuity and local details remains an important challenge in current crack segmentation research.

### 2.2. Visual State Space Models

In recent years, State Space Models (SSMs) have garnered widespread academic attention for their ability to achieve efficient long-range dependency modeling with linear time complexity [20]. By introducing an innovative selective state space mechanism, the Mamba model achieved sequence modeling capabilities far exceeding those of traditional SSMs while maintaining linear computational complexity, offering a highly promising alternative to traditional self-attention structures [21]. Subsequently, pioneering works such as Vision Mamba (ViM) [22] and VMamba [34] extended state space modeling into the 2D visual domain, successfully achieving spatial dependency modeling in images by transforming 2D feature maps into 1D sequences via cross-scanning strategies.

With the rapid advancement of visual state space models, an increasing number of studies have integrated Mamba into various tasks. For example, U-Mamba [35] and SegMamba [36] pioneered the validation of SSMs’ unique advantages in modeling continuous linear structures. SCSegamba [37], proposed by Liu et al., further explored the application of lightweight visual Mamba in crack segmentation by designing a structure-aware state space module that effectively enhanced the model’s perception of crack edge textures and directional structures. Moreover, MambaCrackNet [28] introduced a residual Mamba module, significantly reducing complex background interference and improving the detection rate of micro-cracks; CrackMamba [38] proposed a snake-like scanning strategy, further elevating the model’s representation capacity for complex topological cracks. Despite the strong potential shown by existing visual Mamba methods in long-range dependency modeling, their scanning mechanisms and feature modeling approaches still possess evident limitations, making it difficult to fully adapt to the specific requirements of crack segmentation. Firstly, most current methods employ fixed-direction or parallel cross-scanning strategies for 2D feature serialization, which tends to disrupt original spatial neighborhood relationships when processing cracks, thereby weakening the model’s representation of crack directional continuity and long-range structural associations. Secondly, the original Mamba structure lacks the local spatial inductive bias inherent to convolutional networks; thus, its ability to model fine-grained edge textures and local structural details in complex backgrounds remains constrained.

Explicitly distinguishing from these existing Mamba-based crack segmentation methods, our proposed SGL-Mamba framework establishes a novel paradigm by incorporating a dual-branch Mamba encoder, a frequency-decoupled skip connection, and a deformable large-kernel decoder, specifically tailored to overcome the aforementioned bottlenecks of current visual Mamba models.

Therefore, how to maintain efficient global modeling capabilities while further augmenting the model’s joint representation of crack directional continuity, local boundary details, and robustness to complex backgrounds remains a critical and urgent problem in current visual Mamba-based crack segmentation research. This fundamentally constitutes the core motivation and starting point of our work.

## 3. Methodology

To accurately extract thin, intricate, low-contrast cracks, SGL-Mamba is formulated with highly tailored structural mechanisms. In the encoding stage, a structure-aware scanning mechanism operates in parallel with a local perception pathway to simultaneously capture global topological continuity and fine-grained local details. For feature fusion, a high–low frequency separation enhancement module is designed to explicitly filter out background noise within the skip connections. In the decoding stage, a deformable large-kernel attention mechanism [39] is integrated to dynamically align the spatial receptive field with irregular crack boundaries. These designs collectively reduce background interference and preserve complex crack topologies.

### 3.1. Overall Architecture

**Figure 1:**
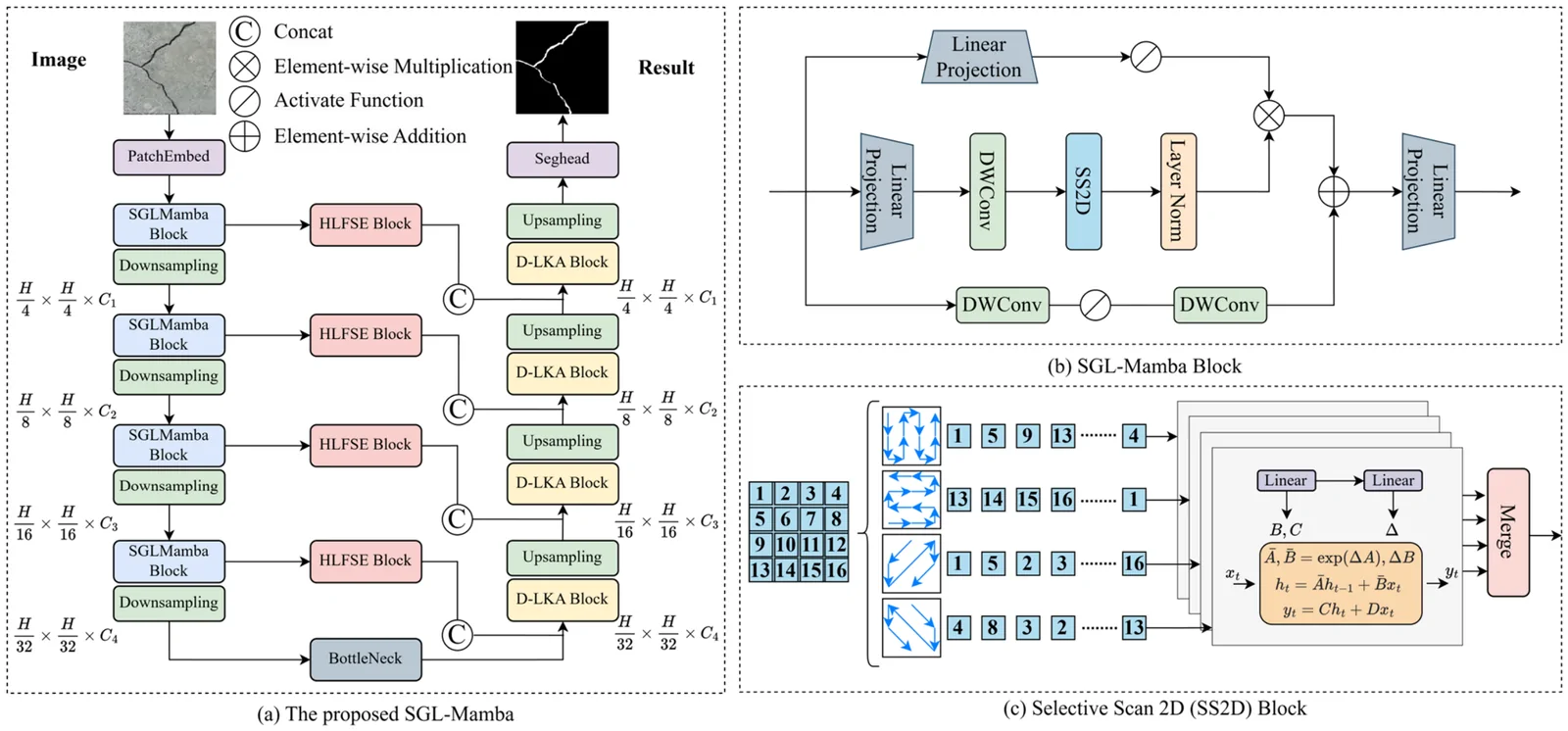
Overall framework of the proposed SGL-Mamba.

To achieve high-precision crack segmentation under complex backgrounds, this paper proposes a novel Structure-Aware Global–Local State Space Network (SGL-Mamba). As illustrated in Figure 1a, the network is constructed based on the classic symmetrical U-shaped encoder–decoder architecture.

Specifically, given an input image $I_{in}\in {\mathbb{R}}^{H\times W\times 3}$, the network first maps it into a high-dimensional feature space utilizing a Patch Embedding module. Subsequently, the feature map sequentially passes through an encoder consisting of four hierarchical stages of SGL-Mamba Blocks to extract and generate multi-scale hierarchical feature representations $F_{i}\in {\mathbb{R}}^{\frac{H}{2^{i+1}}\times \frac{W}{2^{i+1}}\times \left(C\cdot 2^{i-1}\right)}(i=1,2,3,4)$. To bridge the representational divide separating the low-level spatial features of the encoder from the abstract semantics of the decoder, these multi-scale features undergo relay purification via the HLFSE module before being transferred to the corresponding decoding stages. In the decoding phase, the network progressively upsamples the feature maps utilizing the D-LKA module and performs channel-wise concatenation with the refined features output by the HLFSE module. This design enables the decoder to dynamically adapt to the irregular shapes and orientations of cracks while fusing global semantic context with fine-grained spatial details. Finally, a Segmentation Head projects the highest-resolution decoder features into a pixel-level binary probability mask $I_{out}\in {\mathbb{R}}^{H\times W\times 1}$, thereby completing the refined segmentation of the crack regions.

### 3.2. Structure-Aware Global–Local Mamba Block

Cracks typically exhibit highly irregular visual characteristics, such as thin and elongated extensions, multi-directional bending, frequent branching, and blurred boundaries. This poses a dual challenge for crack extraction tasks: on one hand, existing visual Mamba models generally adopt fixed cross-scanning strategies in 2D space, which can easily sever the originally continuous spatial topological structure of cracks, leading to an insufficient capacity for modeling long-range structural dependencies; on the other hand, SSM-based architectures lack the inherent local spatial inductive bias of CNNs, thereby limiting their capability to extract fine-grained edge textures and local details of micro-cracks.

To address the aforementioned issues, we design the SGL-Mamba Block, whose internal architecture is illustrated in Figure 1b. This block comprises two primary learning pathways: the Global Structure-Aware Pathway (GSAP) and the Local Perception Pathway (LPP). The GSAP is specifically designed to meet the requirements of modeling global crack topological continuity; it customizes a four-directional structure-aware scanning strategy tailored to the multi-directional extension characteristics of cracks (Figure 1c) on top of the computational mechanism of the generic 2D Selective Scanning (SS2D) model. Concurrently, the LPP operates in parallel to compensate for the local spatial inductive bias missing in Mamba, specifically focusing on strengthening the extraction of fine-grained edge textures.

Let the input feature of the block be denoted as $X_{i}\in {\mathbb{R}}^{H\times W\times C}(i=1,2,3,4)$. The network first projects it into a high-dimensional hidden space via a linear mapping, and injects an initial local spatial prior using depth-wise separable convolutions:

(1) $$X_{mid}={\mathrm{DWConv}}_{3\times 3}({\mathrm{Linear}}_{mid}(X_{i}))$$

Subsequently, the feature $X_{mid}$ is fed into the SS2D module based on the structure-aware four-directional scanning strategy. To prevent a single scanning path from fragmenting complex crack-connected regions, we unfold the 2D features into four independent 1D sequences along four structured directions (snake scan, column scan, main diagonal scan, and anti-diagonal scan). These four sequences pass through the Selective State Space Model in parallel for long-range sequence modeling and are then inversely restored to 2D spatial shapes and merged via element-wise addition, finally yielding the feature $X_{ssm}$ after Layer Normalization. Concurrently, a gating branch executes linear mapping and SiLU activation on the input $X_{i}$ to dynamically generate attention gating weights G. Element-wise multiplication is then performed between the feature $X_{ssm}$ and the gating weights G to obtain the global feature $X_{global}$. This enables the network to adaptively enhance the activation response of key crack trunks while suppressing background noise. The mathematical expressions of the above process are formulated as follows:

(2) $$X_{ssm}=\mathrm{LayerNorm}(\mathrm{SS}2D(X_{mid}))$$

(3) $$G=\mathrm{SiLU}({\mathrm{Linear}}_{gate}(X_{i}))$$

(4) $$X_{global}=X_{ssm}⊗G$$

where ⊗ denotes the element-wise Hadamard product.

Furthermore, considering the limitations of Mamba in perceiving high-frequency edges and local micro-textures, we design the parallel LPP branch. Operating entirely within the 2D spatial dimension, this pathway utilizes cascaded depth-wise convolutions (DWConv) and point-wise convolutions to thoroughly extract the fine-grained local features of cracks:

(5) $$X_{local}={\mathrm{DWConv}}_{3\times 3}(\mathrm{SiLU}({\mathrm{DWConv}}_{3\times 3}((X_{i}))))$$

Finally, the global feature $X_{global}$, rich in long-range topological continuity, and the local feature $X_{local}$, focusing on edge textures, are aggregated via pixel-level addition. The fused features are then projected back to the original channel dimension C through a linear layer to yield the final output of the block $F_{i}(i=1,2,3,4)$:

(6) $$F_{i}={\mathrm{Linear}}_{out}(X_{global}+X_{local})$$

In summary, while maintaining linear computational complexity, the SGL-Mamba Block effectively preserves the global structural integrity of cracks through the four-directional scanning mechanism of the SS2D submodule. Empowered by the dual support of the local perception branch and the gating mechanism, it significantly improves the model’s feature restoration capability in complex backgrounds and low-contrast scenarios.

### 3.3. High–Low Frequency Separation Enhancement Module

Existing feature concatenation methods easily introduce background redundancy and noise, and struggle to bridge the semantic gap, which often leads to the blurring of the boundary topologies of thin, low-contrast cracks in complex scenes. To address this, we design the HLFSE module. By employing adaptive spectral gain regulation and a cascaded channel-spatial attention mechanism, this module dynamically modulates high- and low-frequency features. It effectively filters out background interference while achieving the precise extraction of weak boundaries in irregular cracks.

**Figure 2:**
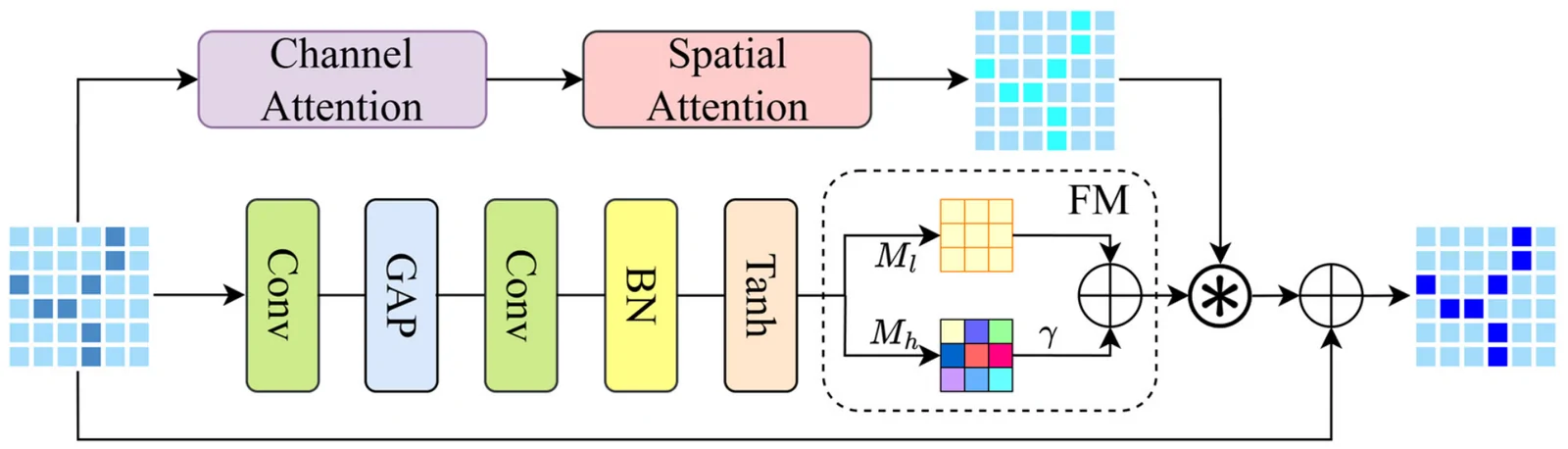
The proposed HLFSE.

As shown in Figure 2, Global Average Pooling and Global Max Pooling are initially applied by the upper branch to the input feature map of the skip connection $F_{i}\in {\mathbb{R}}^{H\times W\times C}$, aiming to aggregate the channel-level semantic correlations across the entire image. A shared Multi-Layer Perceptron (MLP) is then employed to dynamically generate channel calibration weights $W_{c}\in {\mathbb{R}}^{1\times 1\times C}$, which dynamically recalibrate the channel dimension of the input features. Mathematically, this computational step can be expressed as:

(7) $$W_{c}=\sigma (\mathrm{MLP}(\mathrm{GAP}(F_{i}))+\mathrm{MLP}(\mathrm{GMP}(F_{i})))$$

(8) $$F_{c}=F_{i}⊗W_{c}$$

where σ represents the Sigmoid activation function. GAP refers to Global Average Pooling, and GMP refers to Global Max Pooling.

Subsequently, to accurately localize the geometric trajectories of the cracks spatially and suppress background artifacts, Average Pooling, alongside Max Pooling, is executed across the channel dimension of the channel-calibrated features $X_{c}$. The concatenated 2D spatial descriptors are then fed into a 7×7 large kernel convolution to dynamically generate spatial enhancement weights $W_{s}\in {\mathbb{R}}^{1\times H\times W}$. This process is expressed as:

(9) $$W_{s}=\sigma ({\mathrm{Conv}}_{7\times 7}((\mathrm{AP}(F_{c})©\mathrm{MP}(F_{c}))))$$

(10) $$F_{cs}=F_{c}⊗W_{s}$$

where © represents channel concatenation, AP represents Average Pooling, and MP represents Max Pooling. Ultimately, the upper branch outputs high-quality feature maps $F_{cs}\in {\mathbb{R}}^{H\times W\times C}$ that have undergone multi-dimensional spatial and channel pre-calibration, laying a solid foundation for subsequent refined sampling.

Concurrently, while the upper pathway performs feature purification, the lower pathway parallelly computes a dual-frequency adaptive mask tailored to the irregular edges of the cracks. To strike a balance between computational diversity and parameter overhead, we introduce a grouped weight-sharing mechanism. This mechanism divides the features into G groups along the channel dimension, with each group sharing a set of adaptive modulation weights dedicated to a local grid neighborhood (of size K×K).

Initially, local neighborhood features are extracted via 3×3 convolution. Following global average pooling and 1×1 convolution, the hyperbolic tangent (Tanh) activation function is applied to generate the initial dynamic matrix $M\in {\mathbb{R}}^{K\times K\times G}$:

(11) $$M=\mathrm{Tanh}(\mathrm{BN}({\mathrm{Conv}}_{1\times 1}(\mathrm{GAP}({\mathrm{Conv}}_{3\times 3}(F_{i})))))$$

Since the essential difference between distinct crack structures and blurred backgrounds predominantly resides in the high-frequency components of the spectrum, the module introduces a Filter Modulation (FM) operator to spectrally decouple the initial matrix M. This separates it into a low-frequency component $M_{l}$ that retains the DC (Direct Current) components, and a high-frequency component $M_{h}$ that captures specific edges:

(12) $$M_{l}=\frac{1}{K^{2}}E$$

(13) $$M_{h}=M-M_{l}$$

where $E\in {\mathbb{R}}^{K\times K\times G}$ denotes an all-pass matrix with all elements being 1. Subsequently, a set of optimizable channel-level feature enhancement coefficients γ is introduced to apply targeted gain regulation on the high-frequency component, which is rich in boundary details. This is linearly recombined with the low-frequency component to obtain the final refined feature modulation map $\tilde{M}$:

(14) $$\tilde{M}=\gamma \cdot M_{h}+M_{l}$$

Finally, in the spatial aggregation stage, the module applies the dynamic dual-frequency weights $\tilde{M}$ generated by the lower branch to the purified features $F_{cs}$ pre-calibrated by the upper branch. This study adopts a Dilated Sampling mechanism to execute a weighted sum within an expanded, spatially discontinuous neighborhood. For a target pixel in the g-th group at spatial coordinates (h,w), its final output $Y_{i(h,w,g)}$ is defined as:

(15) $$Y_{i(h,w,g)}=\sum_{n=0}^{K-1}\sum_{m=0}^{K-1}F_{{cs}_{\left(h-\left\lfloor \frac{K}{2}\right\rfloor d+md,w-\left\lfloor \frac{K}{2}\right\rfloor d+nd,g\right)}}{\tilde{M}}_{i,j,g}+F_{i(g,h,w)}$$

where $\left\lfloor \cdot \right\rfloor$ denotes the floor operation, and d represents the dilation rate that controls the range of the receptive field.

### 3.4. Deformable Large Kernel Attention

**Figure 3:**
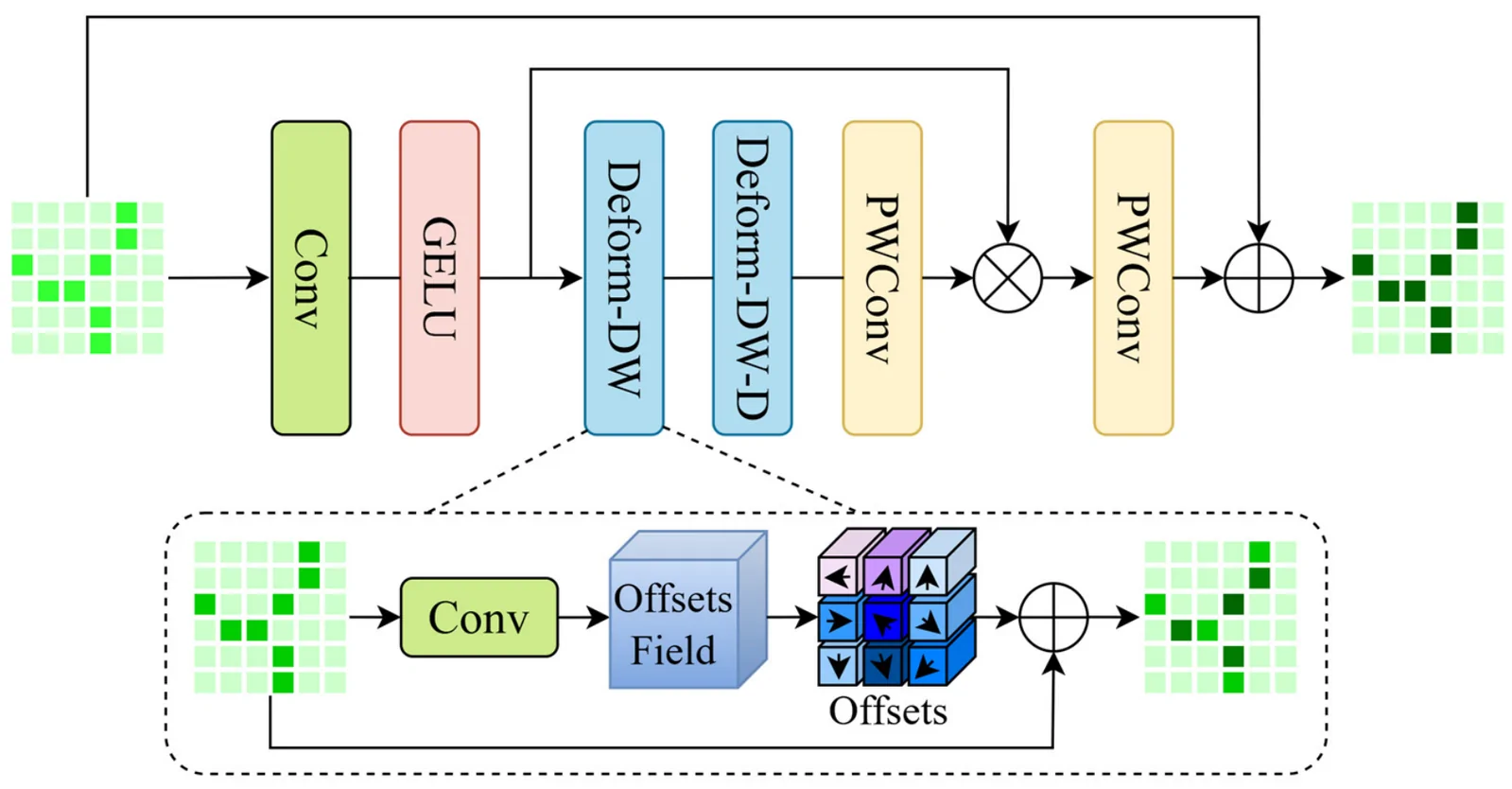
D-LKA Block.

We introduce the D-LKA [39] into the decoder, whose detailed structure is shown in Figure 3. This module integrates Large Kernel Attention (LKA) with Deformable Convolution. It can accurately extract the global topological continuity of cracks with linear computational complexity, while simultaneously prompting the sampling grid to dynamically align with the irregular trajectories of the cracks.

Specifically, for a large convolutional kernel K×K with a target equivalent receptive field, LKA achieves a lightweight decomposition through a sequential combination of depth-wise convolution (DW) due to the introduction of excessive low-frequency background, a depth-wise dilated convolution (DW-D), and a 1 × 1 point-wise convolution. The dimensions of each decomposed kernel satisfy the following equations:

(16) DW=(2d−1)×(2d−1)

(17) $$DW-D=\left\lceil \frac{K}{d}\right\rceil \times \left\lceil \frac{K}{d}\right\rceil$$

where d denotes the dilation rate of the dilated convolution, and $\left\lceil \cdot \right\rceil$ represents the ceiling operation.

To further adapt to the complex geometric deformation characteristics of cracks, the network utilizes an additional convolutional layer to learn deformations from the feature map and generate a spatial offset field, replacing the aforementioned DW and DW-D layers with deformable convolutions. This mechanism allows the sampling grid to dynamically bend and branch along the topological trajectories of the cracks, thereby completely breaking free from the receptive field limitations of fixed regular grids. Let the input of this module be the feature map $F_{i}^{in}\in {\mathbb{R}}^{H\times W\times C}(i=1,2,3,4)$ in the decoder fused with skip connection details, where H and W indicate the spatial dimensions (height and width) of the feature tensor, while C defines its channel depth. The complete forward calculation process of the module is as follows:

(18) $$F^{\prime }=\mathrm{GELU}(\mathrm{Conv}(F_{i}^{in}))$$

(19) $$F_{Attn}={\mathrm{Conv}}_{1\times 1}(Deform-DW-D(Deform-DW(F^{\prime })))$$

(20) $$F_{i}^{out}={\mathrm{Conv}}_{1\times 1}(Attn⊗F^{\prime })+F_{i}^{in}$$

where $F^{\prime }$ is the preprocessing result of the input feature after convolution and GELU activation; Deform−DW represents the deformable depth-wise convolution, and Deform−DW−D denotes the deformable depth-wise dilated convolution, which together constitute the adaptive large-kernel sampling branch; $F_{Attn}$ is the attention weight map output by the module, used to reinforce effective crack features and suppress background noise; the element-wise Hadamard Product achieves the weighted modulation of features by attention.

The final residual connection guarantees the lossless transmission of the original fine crack features, effectively preventing the dissipation of feature information during the decoder’s upsampling process. Prior to the upsampling operation at each stage of the decoder, this paper first enhances the fused features through the D-LKA module: on one hand, it leverages the large-kernel structure to model the global continuous topology of cracks, preventing the occurrence of crack fragmentation after upsampling; on the other hand, it precisely captures the irregular edges and micro-branches of cracks through deformable sampling, significantly improving the localization accuracy of segmentation boundaries. The features enhanced by D-LKA are then fed into the upsampling layer for spatial resolution recovery, thereby effectively compensating for the performance shortcomings of conventional decoders in thin, elongated, and irregular crack segmentation tasks.

### 3.5. Loss Function

Considering the severe class imbalance issue inherent in crack segmentation tasks, we construct a hybrid loss function that combines Binary Cross-Entropy (BCE) loss and Dice loss. The Dice loss directly optimizes the overlap between the predicted segmentation mask and the ground truth (GT) labels, possessing a natural robustness against extreme class imbalance. Concurrently, the BCE loss penalizes pixel-level classification errors, helping refine the boundary details of cracks. The hybrid loss function is defined as:

(21) $$L_{Dice}=1-\frac{2\sum_{j=1}^{N}p_{j}\hat{p_{j}}+\epsilon }{\sum_{j=1}^{N}p_{j}+\sum_{j=1}^{M}\hat{p_{j}}+\epsilon }$$

(22) $$L_{BCE}=-\frac{1}{N}\sum_{j=1}^{N}\left[p_{j}\log\left(\hat{p_{j}}\right)+\left(1-p_{j}\right)\log\left(1-\hat{p_{j}}\right)\right]$$

(23) $$L=\alpha \cdot L_{Dice}+\beta \cdot L_{BCE}$$

where N represents the total number of pixels in a batch, $p_{j}\in \left\{0,1\right\}$ denotes the binary GT label of the j-th pixel (1 for crack, 0 for background), $\hat{p_{j}}\in \left[0,1\right]$ is the predicted crack probability of the j-th pixel, and ϵ=1e−5 is a smoothing factor used to prevent zero-division errors.

## 4. Experiments

### 4.1. Datasets

(1) Crack500: The Crack500 dataset [15] is a classic asphalt pavement crack dataset containing images captured by smartphones in real-world scenarios. The original image resolution is 640 × 360 pixels. To meet the training requirements of deep learning models, the original 500 images were augmented to 3368 images using data augmentation techniques. All images in this dataset are provided with pixel-level binary annotation masks.
(2) DeepCrack: The DeepCrack dataset [12] is highly recognized for its diversity and broad representativeness. It contains 537 RGB images covering typical surface materials such as cement, brick, and asphalt, all with a uniform size of 544 × 384 pixels. Notably, this dataset includes a substantial number of crack samples under extreme and complex conditions, primarily featuring fine cracks, wide cracks, stain interference, and blurred semantic boundaries.
(3) CrackMap: The CrackMap dataset [40] is specifically constructed for road crack segmentation research, consisting of 120 RGB road crack images with a size of 256 × 256 pixels. The image scenes predominantly encompass asphalt, concrete, and brick surfaces. In particular, this dataset focuses on presenting highly irregular, extremely fine, and elongated topological crack networks.

### 4.2. Implementation Details

The SGL-Mamba developed in this study is built upon the PyTorch (PyTorch 1.13.1 and CUDA11.8) framework and trained using an NVIDIA RTX A6000 GPU. The detailed parameter settings for model training are summarized in Table 1. To ensure the objectivity and reproducibility of the experimental results, we employed a stratified random splitting strategy to divide the annotated datasets into training, validation, and independent test sets at a ratio of 7:1:2. Data augmentation techniques, including random flipping, random scaling, and random cropping, were applied during training to enhance the model’s robustness. To ensure statistical robustness, the proposed SGL-Mamba and all ablation variants were trained five times using different random seeds. For our proposed method, the quantitative results are reported as the mean performance followed by the standard deviation (mean ± std).

### 4.3. Evaluation Metrics

To comprehensively evaluate the performance and advancement of our introduced SGL-Mamba network, we referred to previous related research and introduced six of the most widely used evaluation metrics in the field of semantic segmentation: Precision, Recall, F1, Intersection over Union (IoU), Optimal Dataset Scale (ODS), and Optimal Image Scale (OIS). Specifically, ODS assesses the model’s overall performance across the entire dataset using a single constant threshold. Conversely, OIS quantifies the extraction capability on individual images by dynamically selecting the most suitable threshold for each. The corresponding mathematical expressions are given below:

(24) $$P=\frac{TP}{TP+FP}$$

(25) $$R=\frac{TP}{TP+FN}$$

(26) $$F1=\frac{2\times P\times R}{P+R}$$

(27) $$ODS=max_{i}\frac{2\cdot P_{i}\cdot R_{i}}{P_{i}+R_{i}}$$

(28) $$OIS=\frac{1}{N}\sum_{k=1}^{N}max_{n}\frac{2\cdot P_{j,k}\cdot R_{j,k}}{P_{j,k}+R_{j,k}}$$

(29) $$IoU=\frac{TP}{TP+FP+FN}$$

### 4.4. Results and Analysis

To comprehensively validate the effectiveness and advancement of the proposed SGL-Mamba network, extensive comparative experiments were conducted on three public crack datasets: Crack500 [15], DeepCrack [12], and CrackMap [40]. Representative crack segmentation models were selected for comparison, including UNet [30], RINDNet [41], DTrCNet [42], CTCrackSeg [43], Crackmer [44], and SimCrack [45]. Most notably, we also selected recent representative Mamba-series models (CSMamba [46], MambaVision [47], and SCSegamba [37]) as core baselines.

#### 4.4.1. Comparison with SOTA Methods on Crack500

Table 2 presents the quantitative evaluation results of various mainstream models on the Crack500 dataset. Compared to other SOTA methods, our model demonstrates competitive performance, achieving ODS, OIS, Precision (P), Recall (R), F1, and mIoU values of 0.7593 ± 0.0037, 0.7679 ± 0.0036, 0.8149 ± 0.0040, 0.7821 ± 0.0037, 0.7982 ± 0.0038, and 0.8011 ± 0.0036, respectively. Specifically, regarding comprehensive evaluation metrics, our method significantly outperforms the second-best model, SCSegamba, with improvements of 4.29% in F1 and 2.33% in mIoU.

**Figure 4:**
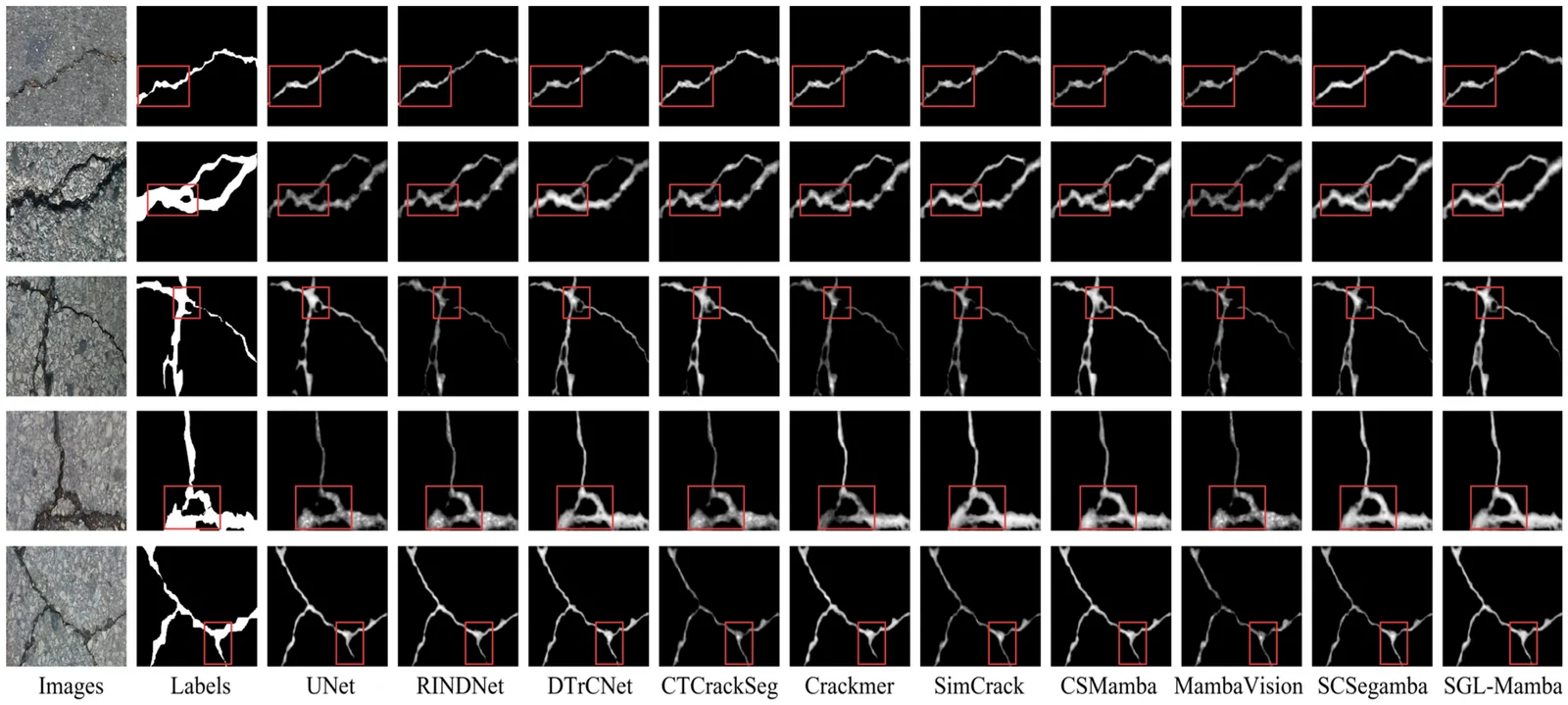
Comparative visualizations of distinct crack instances produced by our model and nine SOTA methods on Crack500. The intricate details are enclosed within red bounding boxes.

Figure 4 illustrates the qualitative visual comparisons of different models. In contrast to alternative frameworks, SGL-Mamba shows better preservation of details when processing areas with distinct crack morphologies. As shown in the first row of Figure 4, when the background interference is weak and the crack lines are simple, the prediction results of all models are within an acceptable range; however, the edge textures extracted by SGL-Mamba align more closely with the GT. Nevertheless, many models, such as RINDNet, Crackmer, and MambaVision, struggle to clearly distinguish ring-shaped cracks (indicated by red rectangular boxes). The second, third, and fourth rows reveal that only SGL-Mamba can successfully detect ring-shaped cracks while preventing the excessive loss of surrounding feature details. In contrast, other networks either miss the cracks entirely or produce indistinct crack feature representations (red boxes). This superiority is primarily attributed to the GSAP utilized in our encoder; its multi-directional and comprehensive scanning mechanism effectively captures anomalous crack features, thereby overcoming the missed detections common in general models when confronting uncommon features. Furthermore, the second and fifth rows demonstrate that SGL-Mamba achieves the best extraction performance at multi-crack intersections. This is because the LPP employed by SGL-Mamba effectively captures the fine-grained edge textures and local spatial details of micro-cracks, delivering superior performance.

#### 4.4.2. Comparison with SOTA Methods on DeepCrack

**Figure 5:**
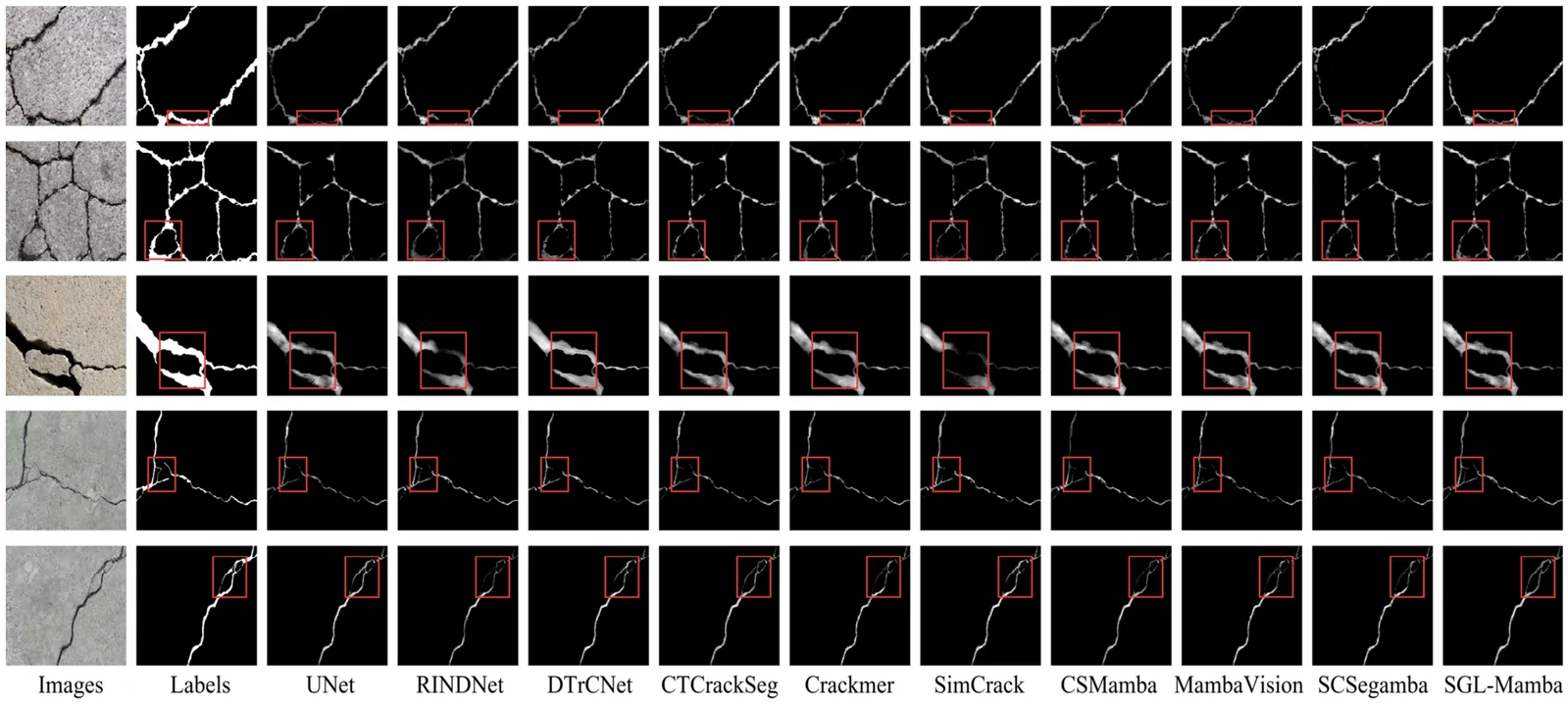
Comparative visualizations of distinct crack instances produced by our model and nine SOTA methods on DeepCrack. The intricate details are enclosed within red bounding boxes.

Table 3 details the quantitative comparison results of different segmentation networks on the DeepCrack dataset. This dataset is challenging as it encompasses high-intensity stain interference, blurred semantic boundaries, and cracks of varying widths. Nevertheless, our model consistently achieved optimal performance across all metrics, with an F1 of 0.9126 ± 0.0038 and an mIoU of 0.9060 ± 0.0034, ranking first among all SOTA methods. As shown in the visual results in Figure 5, our method, SGL-Mamba, produces more precise predictions. The first row of Figure 5 indicates that, with the exception of SCSegamba and SGL-Mamba, other networks fail to detect cracks with relatively complex trajectories (red boxes). The second, fourth, and fifth rows demonstrate that SGL-Mamba successfully extracts key feature information and preserves the relational context of complex topological structures, such as polygons formed by fine cracks. For the third row, SGL-Mamba also maintains high recognition accuracy on relatively thick and wide cracks. This is primarily credited to the precise decoupling and adaptive modulation of high- and low-frequency features by SGL-Mamba’s HLFSE module. By effectively suppressing high-frequency background noise, it directionally enhances the crucial high-frequency texture features of cracks, endowing the network with robust feature discrimination and anti-interference capabilities in complex, low-contrast scenes. In summary, SGL-Mamba demonstrates robust performance in detecting cracks of various shapes and sizes.

#### 4.4.3. Comparison with SOTA Methods on CrackMap

The CrackMap dataset is dominated by highly fine, elongated, and irregular topological crack networks. The quantitative evaluations of the models are presented in Table 4. On this dataset, our method surpasses other comparative methods across most evaluation metrics, achieving an F1 of 0.7828 ± 0.0068 and an mIoU of 0.8145 ± 0.0073, which represent improvements of 1.50% and 0.51%, respectively, over the second-best model, SCSegamba.

**Figure 6:**
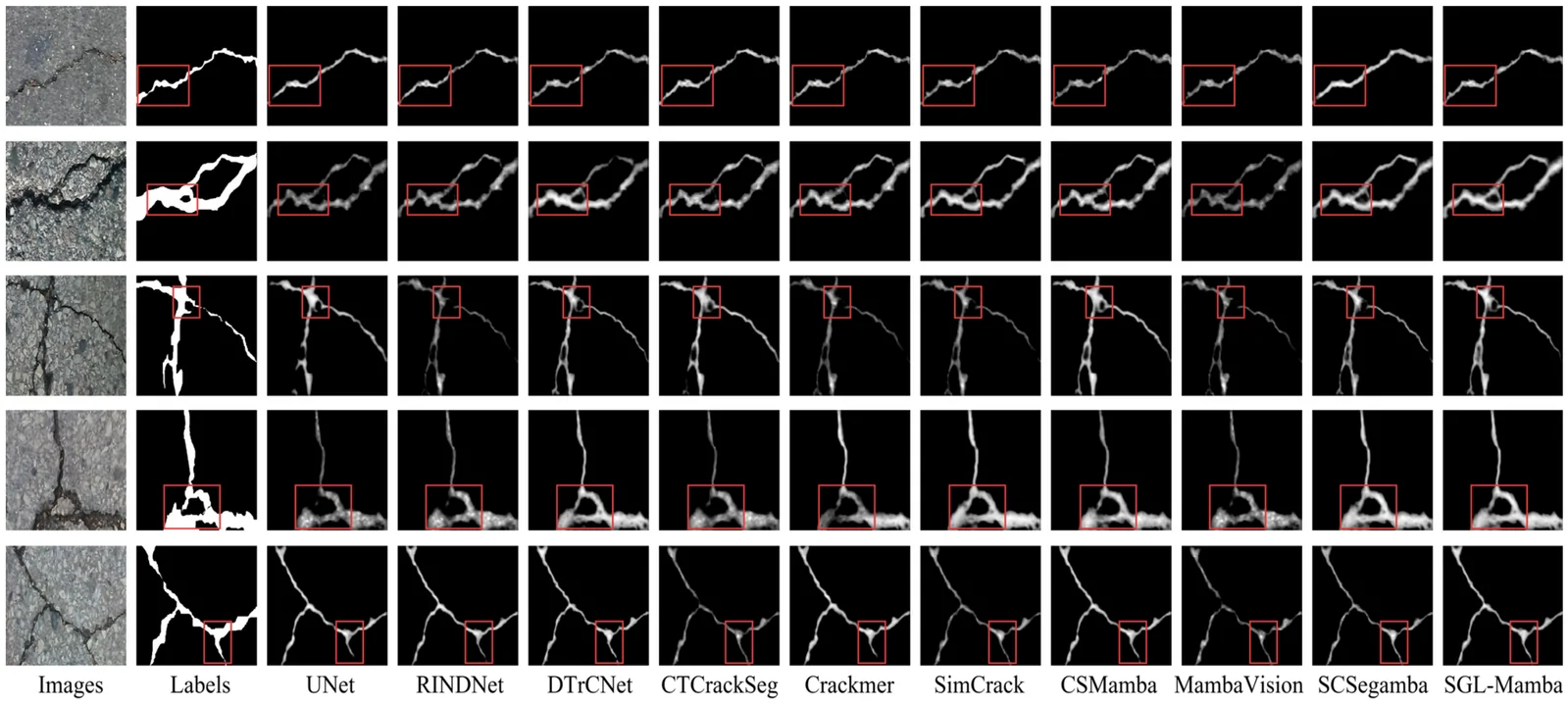
Comparative visualizations of distinct crack instances produced by our model and nine SOTA methods on CrackMap. The intricate details are enclosed within red bounding boxes.

The qualitative comparison in Figure 6 further highlights our model’s superiority in handling complex topologies. Regarding the issue of thin and elongated cracks in this dataset, most models are capable of making predictions, but the actual results vary significantly. Overall, SGL-Mamba’s detection results exhibit the highest concordance with the GT labels (indicated by red boxes in each row). Furthermore, SGL-Mamba successfully predicts the continuous structures where other models (such as RINDNet and MambaVision) experience fragmented missed detections during segmentation (red boxes in the second and third rows). This is mainly due to the D-LKA module in the decoder, which utilizes a deformable large-kernel sampling mechanism to dynamically adapt to the irregular orientations and branches of extremely fine cracks, effectively avoiding fragmentation and missed detections of elongated topological structures.

### 4.5. Ablation Studies

To comprehensively verify each key module of our SGL-Mamba network, this section details a series of exhaustive ablation studies conducted on the Crack500 dataset. We performed quantitative and qualitative analyses across five dimensions: overall network architecture, single core modules, skip connection strategies, loss function weightings, and model complexity. VM-Unet [48] was utilized as the baseline model.

#### 4.5.1. Overall Analysis

To investigate the independent contributions and synergistic effects of the SGL-Mamba encoder, the HLFSE in the skip connections, and the D-LKA decoder module on the overall network performance, we designed a comprehensive ablation study.

**Figure 7:**
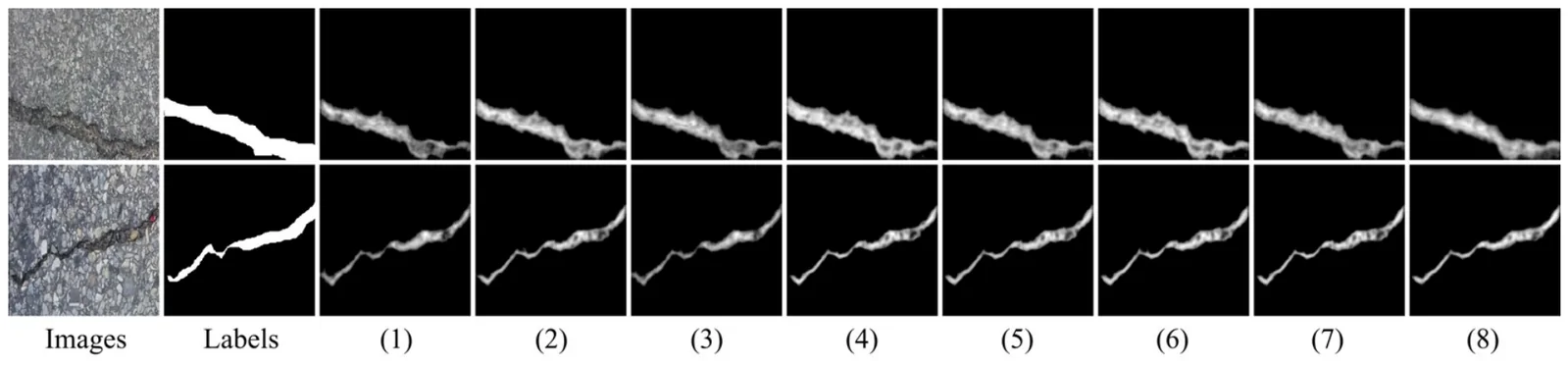
Visual comparison of typical cracks with 8 situations above across Crack500 dataset.

As shown in Table 5, the baseline model (first row) exhibits limited feature extraction capabilities, yielding an F1 and mIoU of only 0.7690 and 0.7757, respectively. When the SGL-Mamba encoder is introduced independently, the model’s mIoU significantly increases by 1.61%, and the F1 improves by 2.53%. This demonstrates the substantial advantage of the structure-aware mechanism in capturing long-range topological dependencies of cracks. The independent introduction of either HLFSE or D-LKA also yields steady performance gains. Notably, when the modules are combined, the model performance is further additively optimized. In particular, when SGL-Mamba, HLFSE, and D-LKA operate synergistically (last row), the network simultaneously accommodates global structural continuity modeling, local anomaly feature extraction, and dynamic adaptation to irregular boundaries, ultimately achieving optimal segmentation performance (F1 of 0.8024 and mIoU of 0.8052). As observed from the qualitative results in Figure 7, the extraction performance of the baseline model (No. 1) is relatively limited. When the SGL-Mamba encoder is introduced independently (No. 2), the global connectivity of the cracks is significantly improved. Similarly, the individual integration of either the HLFSE (No. 3) or the D-LKA decoder (No. 4) yields noticeable visual enhancements. With the combined utilization of multiple modules, the details of the segmentation masks become progressively richer. Ultimately, the full model (No. 8), in which all three modules operate synergistically, achieves the optimal visual performance.

#### 4.5.2. Effectiveness Analysis of SGL-Mamba Block

**Figure 8:**
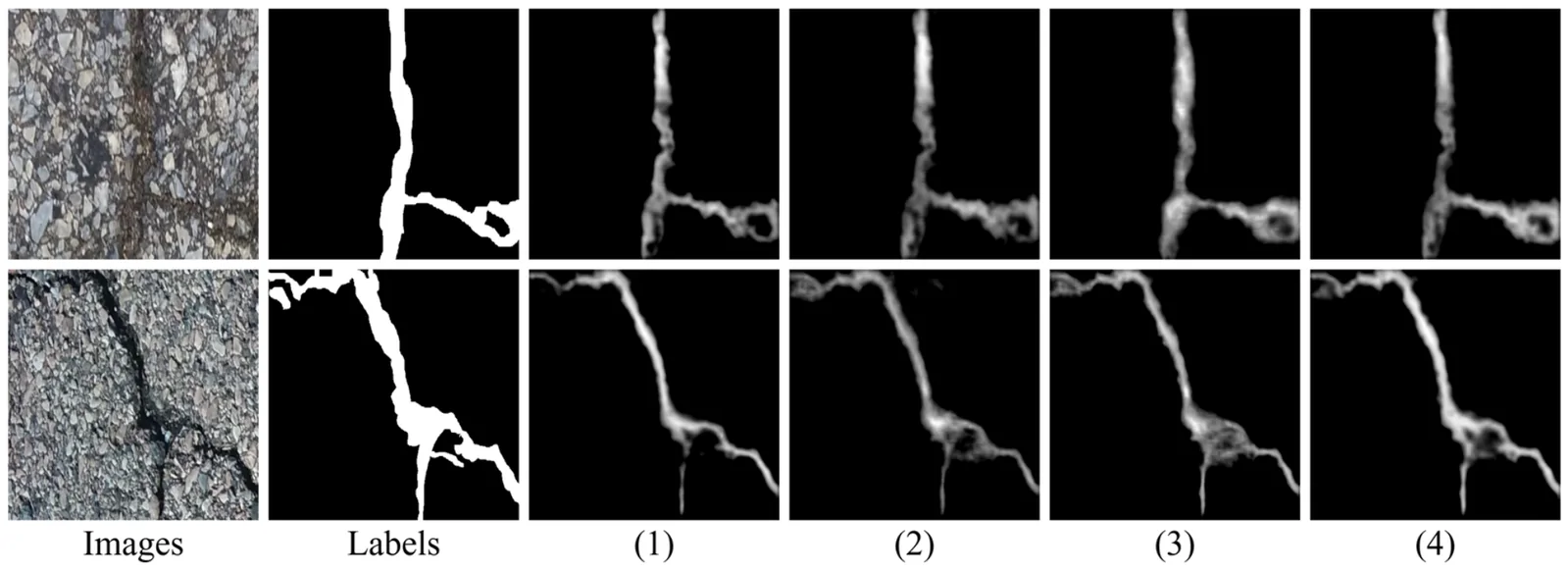
Visual comparison of typical cracks with 4 situations above across Crack500.

The SGL-Mamba Block serves as the core architectural component of the proposed network encoder. To thoroughly validate the effectiveness of its internal mechanisms (GSAP and LPP), we conducted an in-depth internal ablation study. As shown in Table 6, when no improvement components are introduced, we employ the native Mamba block as the baseline model (No. 1). With the selective introduction of GSAP and LPP, the model’s core evaluation metrics exhibit a steady upward trend. Specifically, under the baseline configuration (No. 1), due to the lack of multi-directional scanning spatial dependencies and local spatial priors, the edges of the predicted masks are significantly blurred, accompanied by noticeable topological fragmentation and severe missed detections. Upon introducing only the GSAP branch (No. 2), the multi-directional structured scanning effectively captures global long-range context, seamlessly reconnecting the previously severely fragmented crack trunks. Consequently, the mIoU increases significantly from 0.7866 to 0.7940. This provides compelling evidence of the importance of global context in maintaining the topological integrity of linear features. When solely the LPP branch is introduced (No. 3), the injection of inherent local spatial inductive biases into the 1D sequences notably enhances the model’s ability to delineate fine-grained edge textures, lifting the mIoU by 0.41%. The full SGL-Mamba Block (No. 4), which integrates the dual-branch parallel architecture, achieves a synergistic complementarity between global topological continuity and local boundary details. Further observation of the qualitative visual results in Figure 8 reveals that the fully functional model (No. 4) exhibits the highest consistency with the GT labels. This dual-mechanism fusion design enables the model to not only overcome the topological discontinuity issues caused by traditional fixed scanning strategies but also profoundly fortify the perceptual boundaries of micro-branches when confronting complex and multi-directionally curved cracks. Ultimately, the F1 and mIoU reach their optimal values of 0.8024 and 0.8052, respectively.

#### 4.5.3. Visualization and Interpretability Analysis

**Figure 9:**
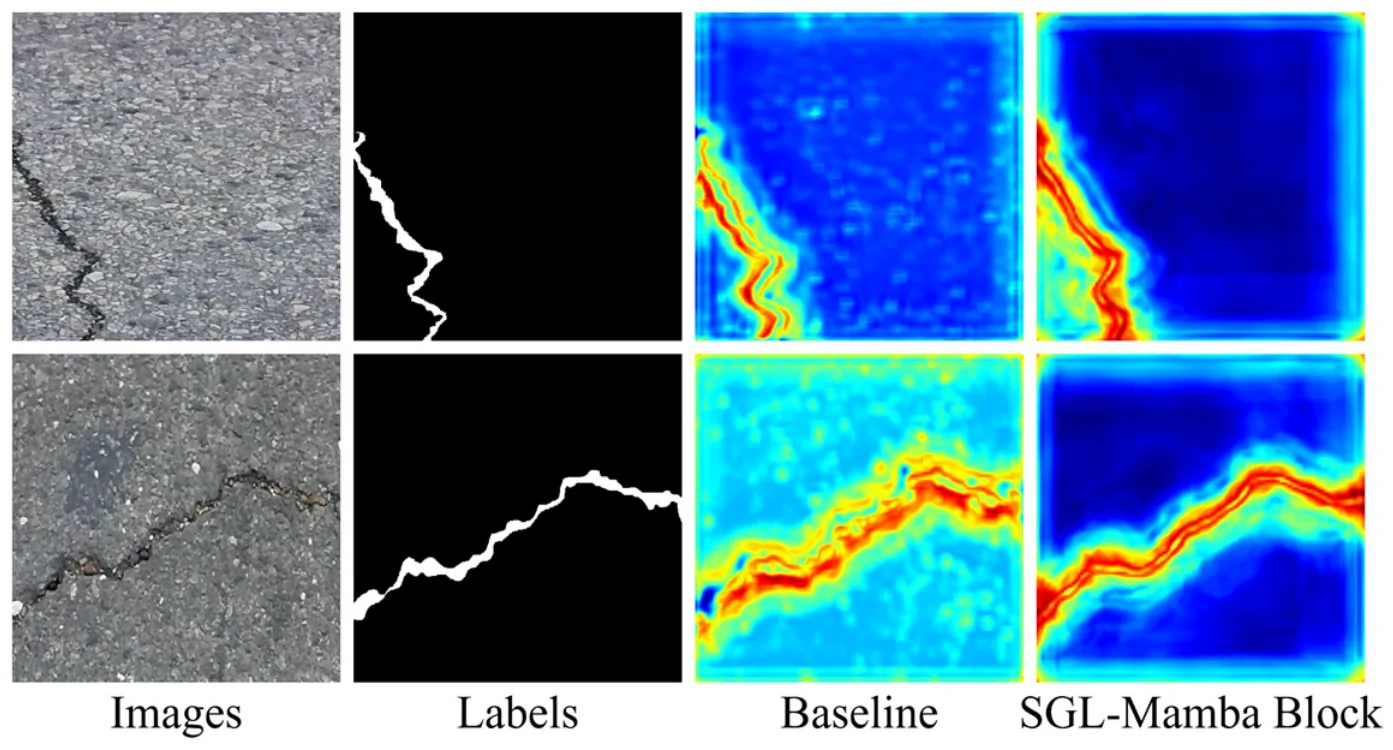
Visual comparison of attention activation maps. The color gradient from blue to red represents the attention activation intensity ranging from low to high.

To further validate the effectiveness of the SGL-Mamba Block, we compare the attention activation maps of the fourth encoder layer between the baseline (VM-UNet) model and our proposed module, as shown in Figure 9. The baseline model’s attention is easily distracted by complex background textures and low-contrast regions, resulting in scattered activations and ambiguous crack boundaries. In contrast, empowered by the GSAP and LPP, the SGL-Mamba Block strongly suppresses background artifacts. The attention weights are highly concentrated on the irregular trajectories of the cracks, proving that our parallel directional scanning and local perception branch can effectively jointly model continuous topological structures and local details.

#### 4.5.4. Effectiveness Analysis of HLFSE

**Figure 10:**
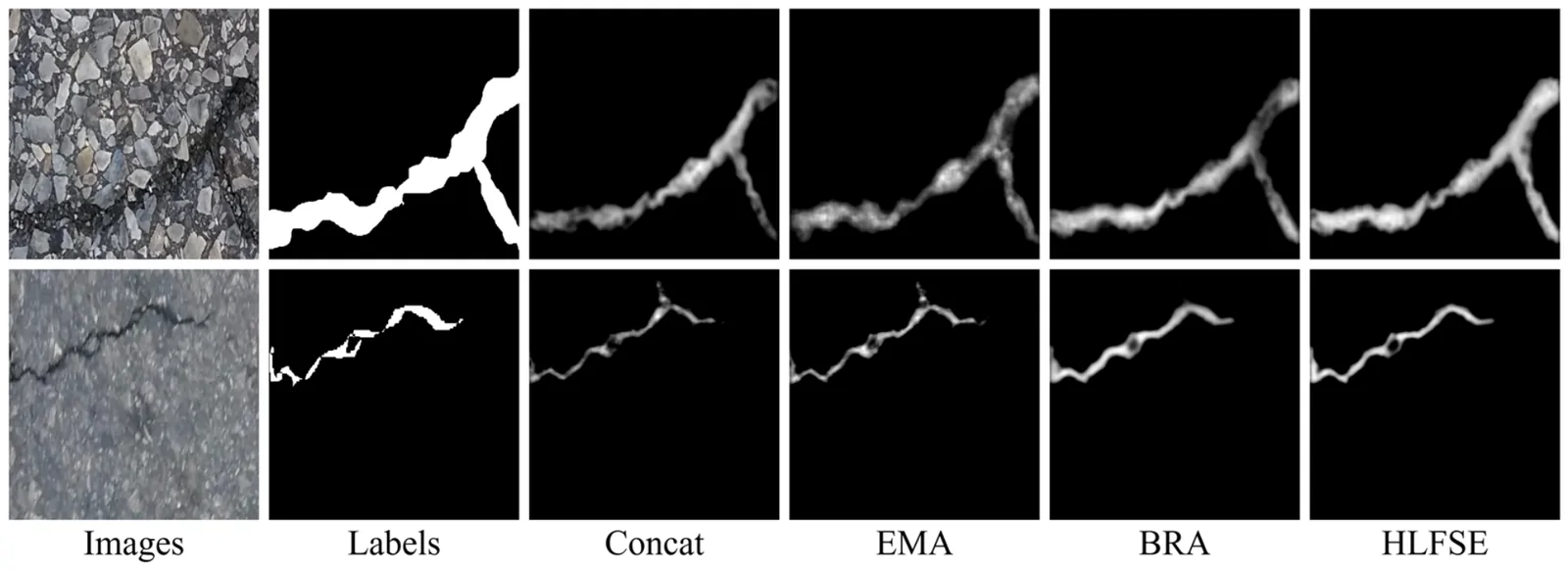
Visually compare typical cracks on Crack500 using four different skip connections.

In U-shape architectures, overcoming the semantic gap to achieve efficient fusion between the low-level details of the encoder and the high-level semantics of the decoder is a critical hurdle for improving segmentation accuracy. Therefore, this paper proposes the HLFSE module. As presented in Table 7, we compared different skip connection strategies. When no feature enhancement strategy is used and direct channel concatenation is applied, the model’s performance is significantly suppressed due to the introduction of excessive high-frequency background noise and semantic redundancy, yielding an mIoU of only 0.7980. After introducing the EMA [49] and BRA [50] mechanisms, although attention reconstruction raises the mIoU to 0.8018 and 0.8036 respectively, they still face challenges with the incomplete detection of complex feature edges. In contrast, by adopting the proposed HLFSE module, the network successfully filters out harmful noise in the skip connections and directionally amplifies the high-frequency texture information of crack edges through adaptive decoupling and precise modulation of high- and low-frequency components. This elevates the F1 and mIoU to an optimal 0.8024 and 0.8052, respectively. Examining the qualitative visual effects in Figure 10, direct concatenation results in severe visual blurring and local topological fragmentation at the predicted crack edges, accompanied by prominent background noise mis-segmentations. With the EMA and BRA modules, these weaknesses are mitigated to some extent, and the main structural skeleton of the cracks becomes clearer. However, when processing complex branches and terminal spurs of micro-cracks, these mainstream attention methods still suffer from feature loss and poor topological connectivity due to their lack of explicit, directional enhancement of frequency-domain features. In comparison, with the HLFSE module, the model precisely restores the micro-branches and high-frequency edge details along the crack trajectories.

Overall, the highly refined edges in the qualitative visualization resonate with the comprehensive lead in quantitative metrics, robustly validating the effectiveness of the HLFSE module.

#### 4.5.5. Hyperparameter Sensitivity Analysis

To provide deeper insight into the design choices of the proposed framework, we conducted a hyperparameter sensitivity analysis on the Crack500 dataset. Since the receptive field size is the most critical factor in balancing the extraction of long-range continuous topologies and the suppression of background noise, we specifically focused on the equivalent kernel size of the D-LKA module in the decoder.

As detailed in Table 8, we evaluated the model with equivalent kernel sizes of K=11,15,21 and 31. The results indicate a clear performance trajectory. Initially, as the kernel size increases from 11 to 21, the comprehensive metrics steadily improve, reaching an optimal F1 score of 0.8024 and an mIoU of 0.8052 at K=21. This demonstrates that an appropriately enlarged spatial receptive field effectively aids the network in capturing the global topological continuity of fine and elongated cracks, seamlessly reconnecting fragmented segments.

However, when the kernel size is excessively expanded to K=31, the performance exhibits a noticeable decline, with the F1 score dropping to 0.7887 and mIoU to 0.7919. This degradation occurs because an overly massive receptive field inevitably introduces excessively complex background artifacts into the sampling grid, which overshadows the fine-grained crack features and leads to structural mis-segmentation. Therefore, setting the equivalent kernel size to K=21 achieves the optimal dynamic equilibrium between structural boundary-refinement and background-noise suppression.

**Figure 11:**
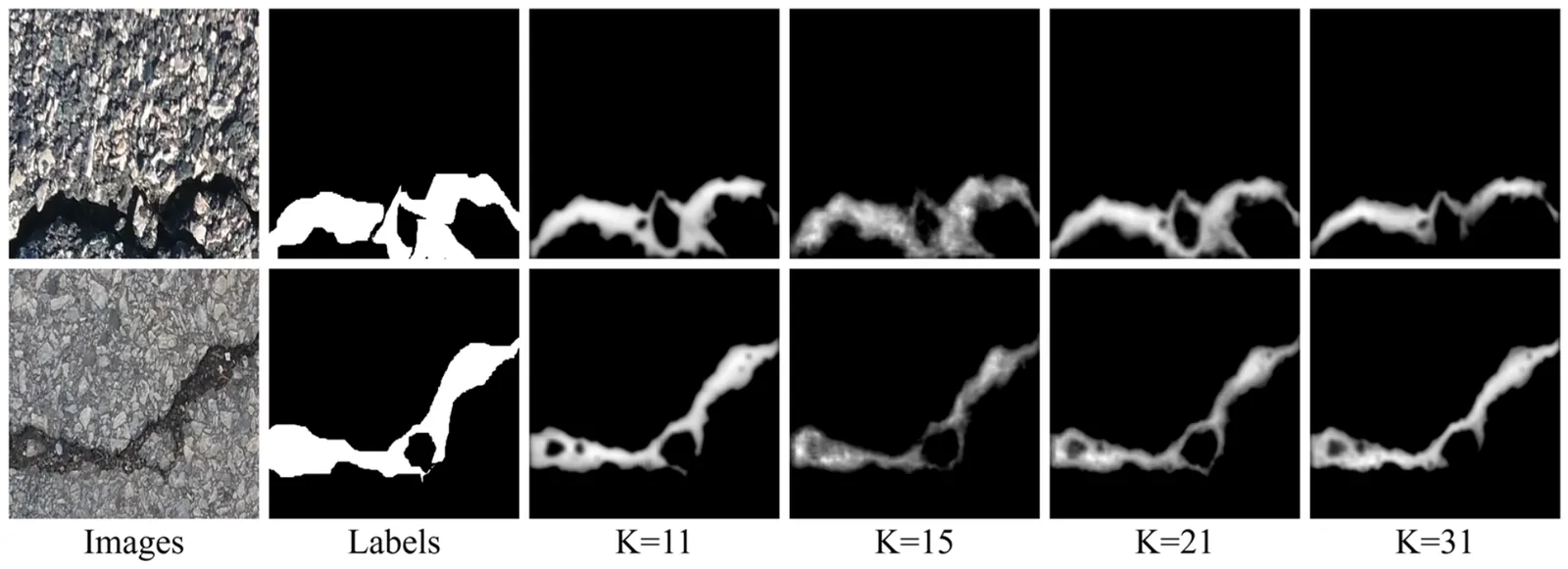
Qualitative visual comparison of crack segmentation results across different equivalent kernel sizes in the D-LKA module.

Figure 11 presents a qualitative visual comparison of the segmentation masks generated under varying equivalent kernel sizes. As observed in the visual results, a smaller receptive field (K=11) struggles to fully grasp the complex topological structures of the cracks. This results in blurred edge delineations and inadequate representation of intricate local features. While K=15 offers slight improvements, the optimal balance is unequivocally achieved at K=21. At this setting, the model perfectly delineates the sharp boundaries and accurately captures both the global continuous skeleton and the fine-grained local topology. Conversely, when the receptive field is excessively expanded to K=31, a noticeable loss of fine topological details occurs. The massive kernel incorporates excessive surrounding background artifacts, causing the intricate loop structure to become distorted and over-smoothed. This visual evidence perfectly aligns with our quantitative data, firmly justifying the selection of K=21 as the optimal configuration for the D-LKA module.

#### 4.5.6. Effectiveness Analysis of Loss Function

**Figure 12:**
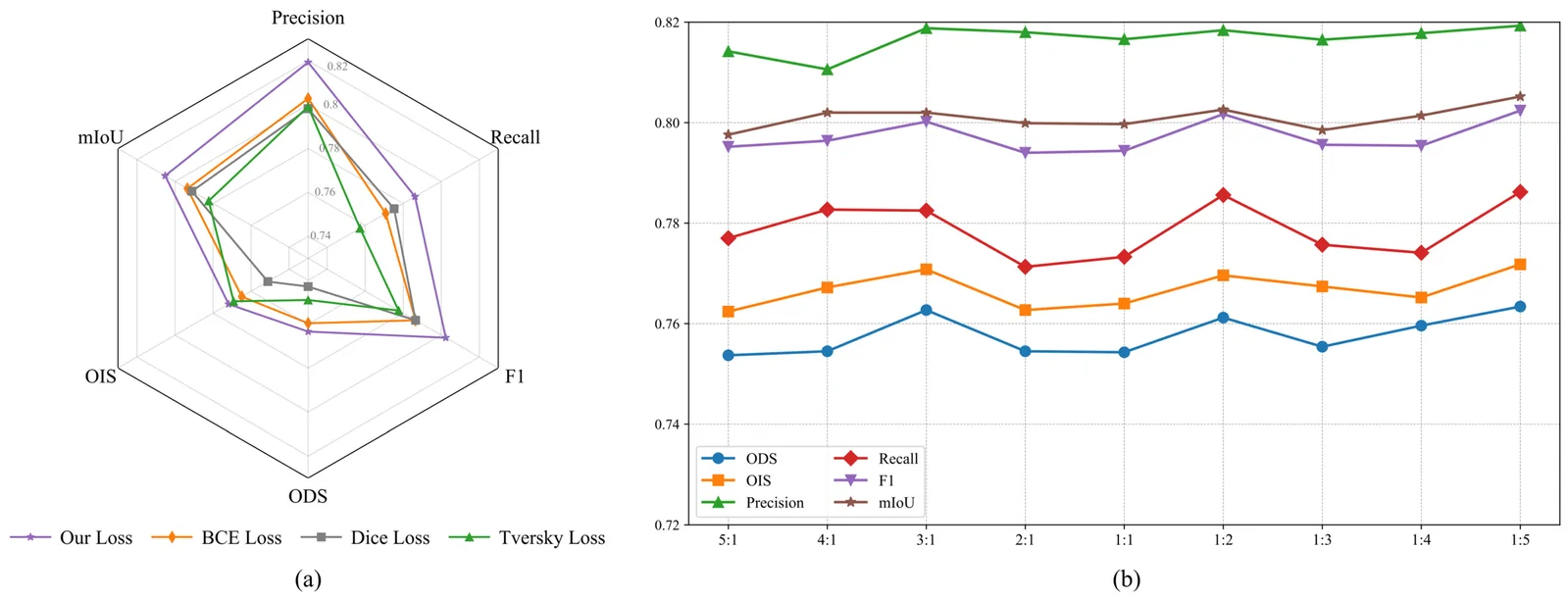
(**a**) Sensitivity analysis of different Loss Functions. (**b**) Sensitivity analysis of different weight ratios between Dice Loss and BCE Loss on the Crack500 dataset.

Given the severe class imbalance between the cracks and the background in crack imagery, we investigated the performance of the model under various loss function configurations. As illustrated in Figure 12a, this study employs a hybrid-driven strategy combining BCE loss and Dice loss. It can be observed that although the standalone BCE loss imposes an effective penalty on pixel-level boundaries, it yields a smaller envelope area because it fails to address the severe class imbalance. Utilizing the Dice loss alone easily drives the model into local optima, making it difficult to preserve intricate edge structures. Conversely, while the comparative Tversky loss mitigates the imbalance to some extent, its performance across various metrics demonstrates a lack of comprehensive balance. In contrast, our proposed hybrid loss strategy exhibits the largest envelope area in the radar chart, comprehensively outperforming other individual or conventional loss functions across all six core evaluation metrics, including Precision, Recall, F1, and mIoU.

Concurrently, to determine the optimal supervisory weight ratio, a series of sensitivity ablation experiments were performed. As shown in Figure 12b, the weight ratio between the Dice loss and BCE loss was systematically varied from 5:1 to 1:5. The experimental results indicate that when the Dice loss dominates the supervision (e.g., at a ratio of 3:1), the model is prone to converging to local optima, exhibiting poor robustness against severely imbalanced data. As the weight of the BCE loss gradually increases, the model becomes highly sensitive to the overlap optimization of the overall crack structure. When the ratio reaches 1:5, the model strikes the optimal dynamic balance between boundary-refinement penalization and imbalance-resistant overlap optimization, with all evaluation metrics reaching their peaks.

### 4.6. Efficiency Analysis

**Figure 13:**
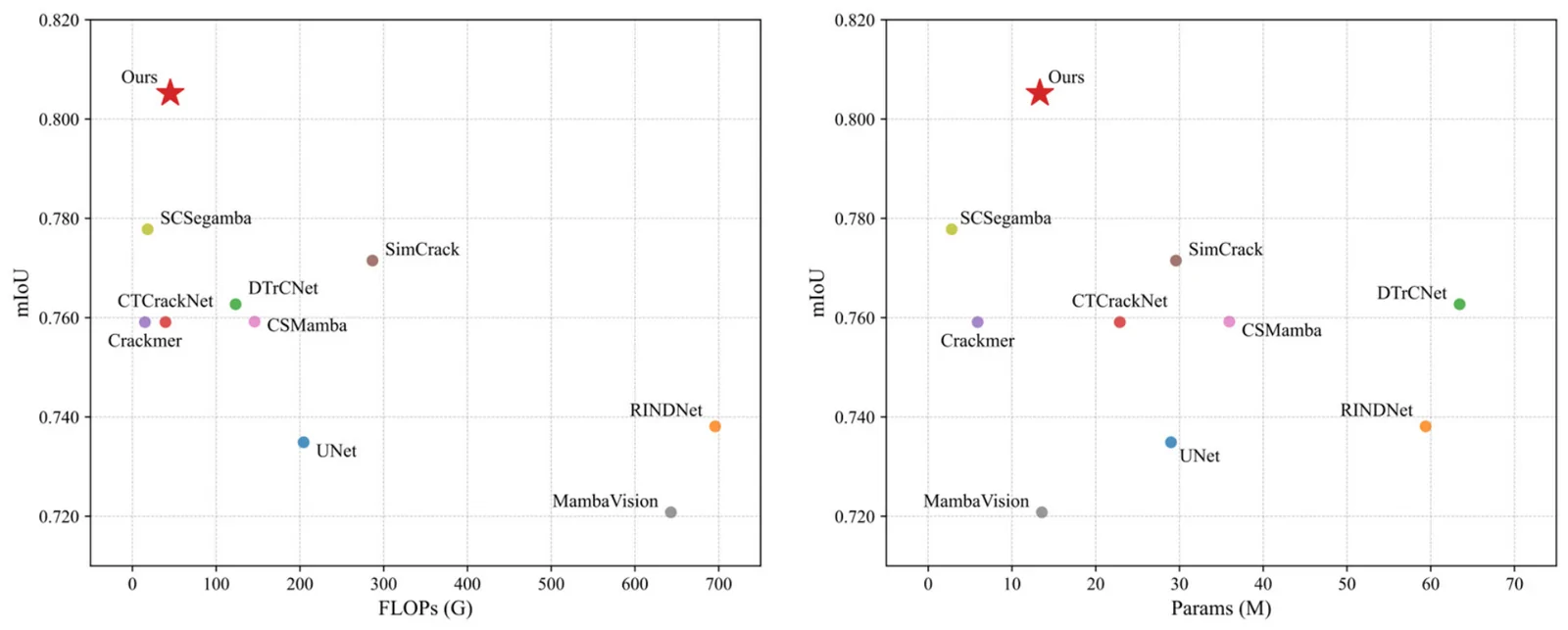
Comparison of complexity with 9 SOTA methods.

Figure 13 illustrates the complexity comparison between the proposed SGL-Mamba and recent mainstream SOTA methods. It is evident that although models like MambaVision and RINDNet possess high theoretical performance, their prohibitive computational burdens severely limit their practical deployment feasibility. Conversely, through the introduction of lightweight deformable large kernel decomposition and state space mechanisms, the proposed method achieves segmentation accuracy far surpassing that of heavyweight networks with an extremely low overhead. To explicitly demonstrate the computational cost introduced by the proposed modules, we incrementally evaluated the model’s parameters and FLOPs. Employing the SGL-Mamba Block independently in the encoder yields a network with 11.84 M parameters and 37.30 G FLOPs. The further integration of the HLFSE module introduces only a marginal overhead, bringing the metrics to 12.07 M parameters and 37.46 G FLOPs. Ultimately, integrating the D-LKA decoder to formulate the complete SGL-Mamba architecture results in a total of 13.54 M parameters and 45.34 G FLOPs. Compared to Crackmer (14.94 G FLOPs) and SCSegamba (18.16 G FLOPs), which are also lightweight architectures, our model incurs a marginal increase in computational cost while achieving the highest performance in key accuracy metrics such as F1 and mIoU, achieving a favorable balance between precision and efficiency.

To further validate the practical deployment capability of the proposed model, we evaluated the empirical runtime metrics of SGL-Mamba. The inference tests were conducted using an input resolution of 512 × 512 and a batch size of 4. Under this configuration, SGL-Mamba achieves an inference speed of 34.70 FPS and a single-image latency of 23.14 ms, while requiring a peak GPU memory consumption of only 1668 MB. These newly reported runtime metrics serve as direct evidence that SGL-Mamba independently meets the real-time processing and low-memory requirements for practical infrastructure health monitoring.

## 5. Discussion

### 5.1. Robustness Under Challenging Imaging Conditions

In practical infrastructure health monitoring, optical sensors often capture images under suboptimal environmental conditions. To comprehensively evaluate the robustness of the proposed SGL-Mamba, we conducted additional perturbation experiments on the Crack500 dataset. Specifically, we designed four evaluation scenarios: (1) No perturbation, (2) Illumination variation, (3) Gaussian noise, and (4) a combined degradation of Illumination variation and Gaussian noise. The models were directly evaluated on these degraded images without any fine-tuning.

The quantitative results are summarized in Table 9. As expected, the introduction of visual perturbations leads to a degradation in segmentation performance. Notably, Gaussian noise causes a more noticeable performance drop than illumination variation, as noise directly corrupts the high-frequency structural details of crack boundaries. However, even under the most severe combined degradation condition, SGL-Mamba maintains a highly competitive performance, achieving an F1 score of 0.7767 and an mIoU of 0.7815. This graceful degradation curve strongly validates the environmental robustness of our architecture. Architecturally, this stability is primarily attributed to the HLFSE module, which adaptively decouples frequency components to filter out severe background noise while amplifying critical edge responses. Furthermore, the D-LKA decoder dynamically adapts its sampling grid to preserve the topological continuity of cracks, ensuring reliable extraction even when image quality is significantly compromised.

### 5.2. Analysis of Challenging Cases

**Figure 14:**
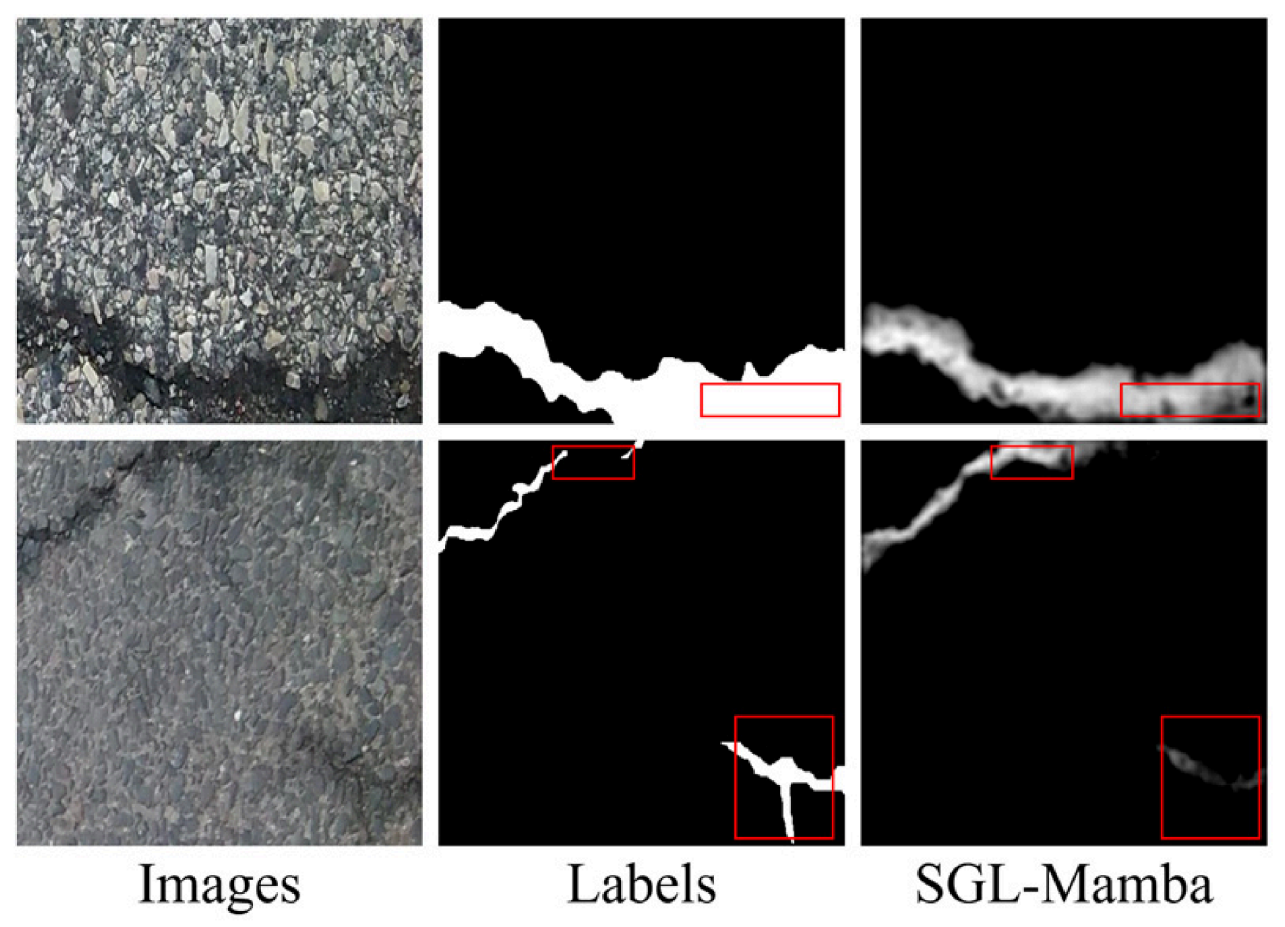
Visualizations of challenging cases and suboptimal predictions on the Crack500 dataset. Red boxes highlight the regions with suboptimal segmentation performance.

While the proposed SGL-Mamba achieves SOTA performance across multiple datasets, it still faces challenges in certain extreme and highly complex scenarios. Figure 14 illustrates representative challenging cases where the model yields suboptimal predictions on the Crack500 dataset. As shown in the first row, in scenes with severe background interference, such as dense gravel textures that closely resemble crack edges, the model may produce blurred boundaries or slight false positives, struggling to sharply isolate the crack from the background noise. Furthermore, as depicted in the second row, when dealing with extremely fine cracks or low-contrast topological segments, the proposed method occasionally suffers from missed detections and fragmented predictions (highlighted by the red boxes).

### 5.3. Mechanistic Analysis of SGL-Mamba

From both architectural and feature-learning perspectives, the superior performance of SGL-Mamba is a direct result of its specialized structural mechanisms. Architecturally, the GSAP utilizes multi-directional scanning to maintain long-range crack continuity without breaking spatial topology, explaining the significant reduction in fragmented predictions observed in Section 4.4. Concurrently, the LPP acts as a high-frequency compensator, ensuring that fine edge details are preserved. From a feature-learning perspective, the HLFSE module dynamically mitigates the semantic gap by explicitly suppressing high-frequency background noise in skip connections. This purified feature space allows the D-LKA decoder to focus its deformable spatial sampling exclusively on irregular crack boundaries. This complementary behavior fundamentally addresses the limitations of standard CNNs and fixed-scan Mamba models in complex and low-contrast scenes.

### 5.4. Limitations and Future Work

As shown in Figure 14, these limitations indicate that while the HLFSE and D-LKA modules significantly improve overall structural continuity, the model’s sensitivity to ultra-fine structural details under heavy background noise requires further enhancement. To address these issues, our future work will focus on exploring cross-dataset generalization by testing the model on additional diverse real-world crack datasets, alongside exploring semi-supervised learning and domain adaptation techniques to improve the robustness of edge feature extraction. Additionally, we will investigate uncertainty-aware prediction mechanisms and model pruning or quantization techniques to facilitate more reliable, computationally efficient, and real-time deployment on edge devices for practical infrastructure health monitoring.

## 6. Conclusions

Cracks generally exhibit features such as thin and elongated extensions, low contrast, multi-branching, and irregular topological structures. Motivated by the aforementioned limitations, we introduce an innovative segmentation model named SGL-Mamba. This model integrates a parallel-structured SGL-Mamba encoder, an HLFSE skip connection module, and a D-LKA decoder fused with large kernel convolutions. While maintaining an extremely low parameter count, it significantly enhances the network’s perception and extraction capabilities for irregular crack textures, continuous topologies, and complex boundaries. Experimental results on three public datasets demonstrate that the overall performance of SGL-Mamba surpasses that of existing mainstream methods. Notably, it exhibits exceptional robustness and segmentation accuracy in highly challenging scenarios characterized by severe noise and fine reticular cracks. Specifically, on the Crack500 dataset, the proposed method achieves an F1 of 0.7982 ± 0.0038 and an mIoU of 0.8011 ± 0.0036, with a parameter count of merely 13.54 M, fully demonstrating its applicability for practical engineering inspection and deployment on edge devices. Future work will introduce semi-supervised learning and domain adaptation techniques, and further explore model pruning and quantization, with the aim of achieving more efficient crack segmentation under constrained edge computing resources.

## Figures and Tables

**Table 1:** The parameter settings.

Item	Setting
Epoch	50
Batch size	4
Optimizer	AdamW
Initial learning rate	5 × 10^−4^
Minimum learning rate	1 × 10^−6^
Learning rate decay type	PolyLR
GPU memory	48 GB
Image size	512 × 512
Loss function	BCE + Dice
Data augmentation	Random flip, random scale, and random crop

**Table 2:** Performance contrast against 9 SOTA methods on the Crack500 dataset. The top-performing metrics are highlighted in boldface, while the runner-up values are indicated with underlines.

Methods	ODS	OIS	P	R	F1	mIoU
UNet	0.6348	0.6444	0.7386	0.7121	0.7251	0.7349
RINDNet	0.6469	0.6483	0.6998	0.7245	0.7119	0.7381
DTrCNet	0.7012	0.7241	0.6527	**0.8280**	0.7357	0.7627
CTCrackSeg	0.6941	0.7059	0.6940	0.7748	0.7322	0.7591
Crackmer	0.6933	0.7097	0.6985	0.7572	0.7267	0.7591
SimCrack	0.7127	0.7308	0.7093	0.7984	0.7516	0.7715
CSMamba	0.6931	0.7162	0.6858	0.7823	0.7315	0.7592
MambaVision	0.6330	0.6377	0.5148	0.6801	0.5860	0.7208
SCSegamba	0.7244	0.7370	0.7270	0.7859	0.7553	0.7778
Ours	**0.7593 ± 0.0037**	**0.7679 ± 0.0036**	**0.8149 ± 0.0040**	0.7821 ± 0.0037	**0.7982 ± 0.0038**	**0.8011 ± 0.0036**

**Table 3:** Performance contrast against 9 SOTA methods on the DeepCrack dataset. The top-performing metrics are highlighted in boldface, while the runner-up values are indicated with underlines.

Methods	ODS	OIS	P	R	F1	mIoU
UNet	0.8858	0.8942	0.8982	0.9008	0.8995	0.8984
RINDNet	0.8616	0.8928	0.8549	0.8692	0.8620	0.8776
DTrCNet	0.8473	0.8512	0.8905	0.8251	0.8566	0.8661
CTCrackSeg	0.8819	0.8904	0.9011	0.8895	0.8952	0.8925
Crackmer	0.8712	0.8785	0.8946	0.8783	0.8864	0.8844
SimCrack	0.8570	0.8722	0.8984	0.8549	0.8761	0.8744
CSMamba	0.8738	0.8766	0.9025	0.8863	0.8943	0.8863
MambaVision	0.8853	**0.9032**	0.8993	0.8607	0.8796	0.8990
SCSegamba	0.8938	0.8990	0.9097	0.9124	0.9110	0.9022
Ours	**0.8954 ± 0.0036**	0.9006 ± 0.0037	**0.9118 ± 0.0038**	**0.9135 ± 0.0039**	**0.9126 ± 0.0038**	**0.9060 ± 0.0034**

**Table 4:** Performance contrast against 9 SOTA methods on the CrackMap dataset. The top-performing metrics are highlighted in boldface, while the runner-up values are indicated with underlines.

Methods	ODS	OIS	P	R	F1	mIoU
UNet	0.7597	0.7606	0.7424	0.7861	0.7636	0.7988
RINDNet	0.6745	0.6943	0.6023	0.7586	0.6699	0.7425
DTrCNet	0.7328	0.7413	0.6912	0.7681	0.7276	0.7812
CTCrackSeg	0.7289	0.7373	0.6911	0.7669	0.7270	0.7785
Crackmer	0.7395	0.7437	0.7229	0.7467	0.7346	0.7860
SimCrack	0.7559	0.7625	0.7380	0.7672	0.7523	0.7963
CSMamba	0.7371	0.7413	0.7053	0.7663	0.7346	0.7841
MambaVision	0.7208	0.7241	0.6891	0.7668	0.7259	0.7736
SCSegamba	0.7741	0.7766	**0.7629**	0.7727	0.7678	0.8094
Ours	**0.7809 ± 0.0068**	**0.7868 ± 0.0073**	0.7492 ± 0.0069	**0.8194 ± 0.0068**	**0.7828 ± 0.0068**	**0.8145 ± 0.0073**

**Table 5:** Component-wise ablation results for the proposed SGL-Mamba. The top-performing metrics are highlighted in boldface, while the runner-up values are indicated with underlines.

No.	SGL-Mamba Block	HLFSE	D-LKA	ODS	OIS	P	R	F1	mIoU
(1)	✗	✗	✗	0.7108	0.7192	0.7888	0.7502	0.7690	0.7757
(2)	✓	✗	✗	0.7577	0.7711	0.8190	0.7710	0.7943	0.7918
(3)	✗	✓	✗	0.7175	0.7221	0.7813	0.7626	0.7718	0.7770
(4)	✗	✗	✓	0.7211	0.7321	0.7791	0.7840	0.7815	0.7810
(5)	✓	✓	✗	0.7546	0.7717	0.8159	0.7756	0.7952	0.7947
(6)	✓	✗	✓	0.7442	0.7639	0.8088	0.7789	0.7936	0.7980
(7)	✗	✓	✓	0.7331	0.7376	0.7997	0.7529	0.7756	0.7828
(8)	✓	✓	✓	**0.7634**	**0.7718**	**0.8193**	**0.7862**	**0.8024**	**0.8052**

**Table 6:** Ablation study of components within the SGL-Mamba Block. The top-performing metrics are highlighted in boldface, while the runner-up values are indicated with underlines.

No.	GSAP	LPP	ODS	OIS	P	R	F1	mIoU
(1)	✗	✗	0.7369	0.7494	0.7817	0.7575	0.7694	0.7866
(2)	✓	✗	0.7467	0.7551	0.8026	0.7814	0.7919	0.7940
(3)	✗	✓	0.7423	0.7550	0.7889	0.7611	0.7747	0.7907
(4)	✓	✓	**0.7634**	**0.7718**	**0.8193**	**0.7862**	**0.8024**	**0.8052**

**Table 7:** Ablation study of different skip connections. The top-performing metrics are highlighted in boldface, while the runner-up values are indicated with underlines.

Skip Connection	ODS	OIS	P	R	F1	mIoU
Concat	0.7442	0.7639	0.8088	0.7789	0.7936	0.7980
EMA	0.7502	0.7608	0.8178	0.7734	0.7950	0.8018
BRA	0.7630	0.7633	0.8150	0.7824	0.7983	0.8036
HLFSE (ours)	**0.7634**	**0.7718**	**0.8193**	**0.7862**	**0.8024**	**0.8052**

**Table 8:** Sensitivity analysis of different equivalent kernel sizes in the D-LKA module on the Crack500 dataset. The top-performing metrics are highlighted in boldface, while the runner-up values are indicated with underlines.

Kernel Size	ODS	OIS	P	R	F1	mIoU
11	0.7538	0.7607	0.8164	0.7732	0.7942	0.7963
15	0. 7589	0. 7681	0. 8117	0.7 844	0. 7978	0. 8011
21	**0.7634**	**0.7718**	**0.8193**	**0.7862**	**0.8024**	**0.8052**
31	0.7426	0.7513	0.7981	0.7795	0.7887	0.7919

**Table 9:** Robustness evaluation of SGL-Mamba under different challenging imaging conditions on the Crack500 dataset. The top-performing metrics are highlighted in boldface, while the runner-up values are indicated with underlines.

Settings	ODS	OIS	P	R	F1	mIoU
No perturbation	**0.7634**	**0.7718**	**0.8193**	**0.7862**	**0.8024**	**0.8052**
Illumination variation	0. 7551	0. 7638	0. 8114	0.7 763	0.7 935	0.7 974
Gaussian noise	0.7432	0.7509	0.8037	0.7645	0.7836	0.7882
Illumination variation + Gaussian noise	0.7387	0.7461	0.7961	0.7582	0.7767	0.7815

## Data Availability

The datasets used in this work are all publicly available crack segmentation benchmarks. The Crack500 dataset is available at https://github.com/guoguolord/CrackDataset/tree/main/crack500 (accessed on 5 March 2026), the DeepCrack dataset can be accessed at https://github.com/yhlleo/DeepCrack (accessed on 5 March 2026), and the CrackMap dataset is available at https://github.com/ikatsamenis/CrackMap (accessed on 5 March 2026).
